#  A point-prevalence study on community and inpatient *Clostridioides difficile* infections (CDI): results from Combatting Bacterial Resistance in Europe CDI (COMBACTE-CDI), July to November 2018

**DOI:** 10.2807/1560-7917.ES.2022.27.26.2100704

**Published:** 2022-06-30

**Authors:** Virginie F Viprey, Georgina L Davis, Anthony D Benson, Duncan Ewin, William Spittal, Jon J Vernon, Maja Rupnik, Alice Banz, Florence Allantaz, Philippe Cleuziat, the COMBACTE-CDI National Coordinators, Mark H Wilcox, Kerrie A Davies, on behalf of the COMBACTE-CDI consortium, Members of the COMBACTE-CDI National coordinators, Elena Novakova, Andreas Matussek, Fidelma Fitzpatrick, Anne Simon, Ioana Macovei, Frederic Barbut, Elena Reigadas Ramirez, Efi Petinaki, Ed Kuijper, Nicola Petrosillo, Hanna Pituch

**Affiliations:** 1 Healthcare Associated Infections Research Group, Leeds Institute of Medical Research, University of Leeds, Leeds, United Kingdom; 2 National Laboratory for Health, Environment and Food, Maribor, Slovenia; 3 Faculty of Medicine, University of Maribor, Maribor, Slovenia; 4 European Society of Clinical Microbiology and Infectious Diseases (ESCMID) Study Group for *Clostridioides difficile* (ESGCD); 5 bioMérieux, Marcy L'Etoile, France; 6 The members of the COMBACTE-CDI National Coordinators are listed under Collaborators; 7 Department of Microbiology, Leeds Teaching Hospitals NHS Trust, Leeds, United Kingdom; 8 The members of the COMBACTE-CDI consortium are listed at the end of this article

**Keywords:** *Clostridioides difficile*, hospital, community, diagnosis

## Abstract

**Background:**

There is a paucity of data on community-based *Clostridioides difficile* infection (CDI) and how these compare with inpatient CDI.

**Aim:**

To compare data on the populations with CDI in hospitals vs the community across 12 European countries.

**Methods:**

For this point-prevalence study (July–November 2018), testing sites sent residual diagnostic material on sampling days to a coordinating laboratory for CDI testing and PCR ribotyping (n = 3,163). Information on whether CDI testing was requested at the original site was used to identify undiagnosed CDI. We used medical records to identify differences between healthcare settings in patient demographics and risk factors for detection of *C. difficile* with or without free toxin.

**Results:**

The CDI positivity rate was 4.4% (country range: 0–16.2) in hospital samples, and 1.3% (country range: 0–2.2%) in community samples. The highest prevalence of toxinotype IIIb (027, 181 and 176) was seen in eastern European countries (56%; 43/77), the region with the lowest testing rate (58%; 164/281). Different predisposing risk factors were observed (use of broad-spectrum penicillins in the community (OR: 8.09 (1.9–35.6), p = 0.01); fluoroquinolones/cephalosporins in hospitals (OR: 2.2 (1.2–4.3), p = 0.01; OR: 2.0 (1.1–3.7), p = 0.02)). Half of community CDI cases were undetected because of absence of clinical suspicion, accounting for three times more undiagnosed adults in the community compared with hospitals (ca 111,000 vs 37,000 cases/year in Europe).

**Conclusion:**

These findings support recommendations for improving diagnosis in patients presenting with diarrhoea in the community, to guide good practice to limit the spread of CDI.

## Introduction

*Clostridioides difficile* infection (CDI) is one of the most common healthcare-associated infections, accounting for almost half of all gastrointestinal infections in hospitals in Europe. Based on 2016–17 point prevalence surveys from the European Centre for Disease Prevention and Control (ECDC), an estimated 189,526 (95% confidence interval (CI): 105,154–340,978) cases are reported annually in acute care hospitals, causing considerable morbidity and mortality [1,2]. Factors frequently reported to contribute to the development of CDI in hospitalised patients include antibiotic use, an increasing Charlson comorbidity index score, an age over 65 years, prior CDI and hospital admission [3-6]. As one in four CDI cases diagnosed in the hospital are estimated to originate in the community [7], early detection and treatment for CDI in the community is required to reduce transmission of infection and prevent the need for hospitalisation, thereby reducing the healthcare burden [8]. A meta-analysis reported that the risk of developing CDI in the community was greater in individuals exposed to clindamycin, fluoroquinolones, cephalosporins and penicillins [9]. However, reliance on predisposing risk factors may not be sufficient to recognise which patients in the community should be tested for CDI. Community cases of CDI have a mean age below 65 years and are less likely to have been exposed to antibiotics [7,10,11]. Studies from the Netherlands and England report that a third of CDI cases in the community did not have a history of antibiotic use or previous contact with a hospital [12,13]. Infrequent testing of stool samples for CDI in the community means that CDI is often undiagnosed in this setting [8]. For example, general practitioners in the Netherlands and France requested CDI testing for only ca 1/10 stool samples submitted [12,14]. Therefore, while the burden and impact of underdiagnosis of CDI in hospitalised patients is well recognised [15-17], data on the true burden of CDI in the community are scarce.

A key component of CDI surveillance is identification of ribotypes. The increased diversity of *C. difficile* ribotypes in hospital samples across Europe was most recently described in 2012–13, with 027, 001/072, and 014/020 found to be the most prevalent (19%, 11%, and 10%, respectively) [18]. The hypervirulent *C. difficile* ribotype 027 is has been widely shown to cause outbreaks [19]. Lack of CDI testing is thought to contribute to outbreaks, as described in a 2012–13 European point-prevalence study in hospitalised patients, where the highest prevalence of 027 was seen in regions of Europe with low testing rates [8,15,18]. Ribotype 078 is a hyper-virulent lineage increasingly found in community samples [8,20]. Although there are limited data on ribotype diversity in the community in Europe, 078 was one of the top five most prevalent in a surveillance study of community-associated cases of CDI in England in 2011–13, with 002 identified as the most prevalent [21].

In this study, we present results from COMBACTE-CDI (Combatting Bacterial Resistance in Europe-CDI), a multi-centre European-wide project and point-prevalence study aimed at providing comprehensive data on the population with CDI across the whole healthcare system in Europe. The aim of this study was to investigate the difference in the frequency of testing of diarrhoeal samples for CDI concurrently in both the hospital and community, including evaluation of undiagnosed cases and predisposing risk factors for CDI.

## Methods

### Study setting 

The COMBACTE-CDI project aimed to provide a detailed understanding of the epidemiology and clinical impact of CDI across Europe. COMBACTE-CDI is a consortium of CDI experts from eight academic research organisations and six industrial partners (The European Federation of Pharmaceutical Industries and Associations (EFPIA) members). We refer to the study presented here as the ‘COMBACTE-CDI point-prevalence and follow-up study’, to distinguish this work from other investigations by the same consortium.

COMBACTE-CDI recruited hospital/laboratory sites carrying out diagnostic testing of samples from both hospitalised and community patients, following the design of a previous European point-prevalence study in hospitalised patients with diarrhoea (The European, multicentre, prospective, biannual, point-prevalence study of *Clostridium difficile* infection in hospitalised patients with diarrhoea, EUCLID) [15]. Sites were selected to give respective coverage, based on population size, in the countries recruited (one site per 3 million population) and to represent the northern, southern, eastern and western regions of Europe, defined according to the United Nations Geoscheme as determined in the EUCLID study [15]. Twelve countries were recruited, totalling 119 testing sites (North: United Kingdom (UK), Ireland, Sweden; South: Greece, Italy, Spain; East: Poland, Romania, Slovakia; West: Belgium, France, the Netherlands).

### Sample collection 

**Figure 1:**
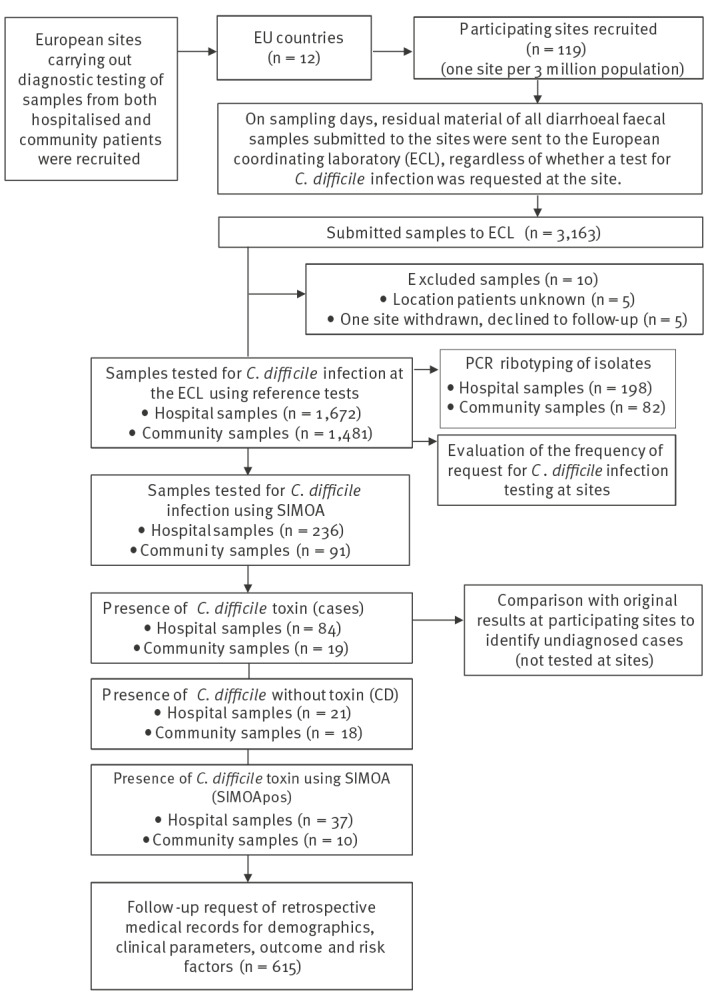
Sample flow-chart of the COMBACTE-CDI point-prevalence and follow-up study, 12 European countries, July–November 2018

Residual diagnostic diarrhoeal faecal samples that had been submitted to the sites for microbiological testing on two non-consecutive sampling weekdays between July and November 2018 were sent to the European Coordinating Laboratory (ECL) in Leeds, UK, for *C. difficile* testing, regardless of whether CDI testing was requested on site (Figure 1). A third sampling day was added for sites in countries with low numbers of samples per day (less than 10 samples per day), i.e. Greece, Ireland, Italy, Poland, Romania, and Slovakia. Participating sites provided information to the ECL on the location of the individual at the time of sampling and whether the sample was tested for CDI by the sites. This information was used to distinguish hospital samples (inpatient setting) from community samples (any non-inpatient setting, e.g. general practitioners), and to calculate the testing rate per country, within the region, and across Europe. Since this was an observational study, the CDI test result at the ECL was not reported back to the sites.

CCNA: cell-cytotoxin neutralisation assay; CTC: cell cytotoxigenic culture; ECL: European coordinating laboratory; SIMOA: ultra-sensitive toxin single molecule array.

Cases were the participant group that was CCNA-positive (presence of *C. difficile* toxin using reference test). CD was the term chosen to describe the participant group that was CTC-positive/CCNA-negative/SIMOA-negative (presence of *C. difficile* without toxin). SIMOApos was the term chosen to describe the participant group that was SIMOA-positive/CCNA-negative (presence of *C. difficile* toxin using a novel test).

### Laboratory procedures

To limit bias caused by the use of potentially different diagnostic methodologies across sites, samples were tested for CDI at the ECL using the cell-cytotoxic neutralisation assay (CCNA), the reference test for the laboratory confirmation of CDI, identifying primarily the presence of free toxin B, but also toxin A [22,23]. The CCNA result was used to calculate the number of CDI cases and the positivity rate per country, within the region, and across Europe. All samples were also tested with cell cytotoxigenic culture (CTC), which detects the presence of *C. difficile* with potential to produce toxin [23]. Samples that were positive by either CCNA or CTC, plus geographically (same site where possible, or same country if not) matched samples that were negative by both assays, were tested with the BioFire FilmArray Gastrointestinal panel (bioMérieux, France) according to the manufacturer’s instructions, to identify alternative pathogens causing diarrhoea and for quantitative detection of toxins A and B by ultra-sensitive toxin single molecule array (SIMOA) technology [24] (performed at bioMérieux). Assays were performed as previously described [24-26]. PCR ribotyping was performed on all isolates following the *C. difficile* Ribotyping Network of England and Northern Ireland protocol [27,28]. The CCNA reference test result at the ECL was compared with the CDI test result at the participating sites to identify the proportion of undiagnosed cases, i.e. positive by CCNA at the ECL (presence of *C. difficile* toxin) but not tested at the submitting site. The clinical data analysis was performed as described below, and to include two new potential outcome groups for CDI; these were samples positive by CTC but negative by both CCNA and SIMOA (presence of *C. difficile* without toxin), and samples positive only by SIMOA but not CCNA (presence of *C. difficile* toxin using novel test only).

### Clinical data and outcomes

Retrospective collection of clinical data from medical records, including follow-up (6 months after sample submission), was provided to the ECL by participating sites using a standardised clinical report form (CRF). The CRF was used to identify baseline and specific characteristics from patients with samples that fitted the following outcome criteria: (i) cases (presence of *C. difficile* toxin; CCNA-positive), (ii) CD (presence of *C. difficile* without toxin; CTC-positive, CCNA and SIMOA-negative), (iii) SIMOApos (presence of low-level *C. difficile* toxin A and/or B; SIMOA-positive, CCNA-negative), and (iv) controls (negative by all assays). Of note, these criteria, used to define three outcome groups and one control group, were only known to the ECL as the CDI test result at the ECL was not reported back to the individual sites. To select an appropriate number of controls from the pool of samples received, and to reduce confounding, the ECL requested medical records data from approximately three control patients from the same geographical location for each patient whose sample was categorised into an outcome group (cases, CD, SIMOApos).

Baseline characteristics were age, gender, healthcare setting (community vs hospital) at the time of sampling, and country name. Specific characteristics (categorical variables) were (i) previous exposure to a healthcare setting (in the previous 6 months before sample submission), (ii) contact with a CDI case or infant (< 1 year of age in the previous 3 months to the sample being taken), (iii) number and (iv) type of antibiotics use in the 12 weeks preceding the sample, (v) known co-morbidities, (vi) clinical parameters (continuous data: white cell count and (vii) number of days of diarrhoea before sample submission; binary data included hospital admission within 30 days of sample submission and 30-day mortality rate.

### Statistical analysis

Statistical analyses of the retrospective patient data were performed with SPSS version 23 (IBM). Upon receipt of the medical records, patients were removed from control groups if they had evidence of CDI within 8 weeks of the sample being taken. Categorical variables and binary data were analysed by chi-squared tests, continuous data were analysed by t-tests, or Mann–Whitney (median) tests. The number of individuals in the outcome (cases, CD, SIMOApos) and control groups who were exposed or not exposed to a categorical variable (listed above in the Clinical Data methods section) was used to calculate an odds ratio (OR), 95% CI, and chi-squared or Fisher’s exact test p values with significance set at 0.05 (when the minimum expected count was less than 5), as a measure of the association between exposure to each of those variables and outcome (cases, CD, SIMOApos).

## Results

The COMBACTE-CDI study recruited 119 sites in 12 countries across Europe, with 3,163 diarrhoeal faecal samples received at the ECL. Ten samples were excluded, including five with patient location unknown at the time of sampling, and five submitted by a site that withdrew from the study at the time of follow-up. Therefore, results are presented from 118 sites with a total of 3,153 samples; 1,672 received from hospital and 1,481 from the community (Figure 1).

### Testing frequency, positivity rate and undiagnosed *Clostridioides difficile* infection cases

The testing and positivity rates in hospital samples were 75.5% (country range: 46.5–100) and 4.4% (country range: 0–16.2), vs 29.9% (country range: 0–100) and 1.3% (country range: 0–2.2) in community samples (Table 1). Co-infections were detected with other gastrointestinal pathogens in only 12/83 (1/84 samples not tested) hospital and 5/19 of community CDI cases (Supplementary Figure S1: Organisms detected by the BioFire FilmArray GI Panel assay). Interestingly, 13/84 of samples positive for CDI at ECL were not tested in hospitals vs 11/19 that were not tested in the community. The possible underlying reasons for not testing those patients with CDI were extracted from the retrospective clinical data, which were obtained for 11/ 13 undiagnosed cases in the hospital and for all 11 undiagnosed cases in the community. After exclusion of patients who had another sample tested within 2 days of the original sample (n = 2 in hospital) and exclusion of paediatric patients (n = 6 in hospital, n = 2 in community), we note that 3/84 hospitalised CDI cases were not tested and therefore truly missed vs 9/19 in the community (Table 1). 

**Table 1:** Testing rate, positivity rate and undiagnosed cases among *Clostridioides difficile* infections across whole healthcare systems, Europe, July–November 2018 (n = 3,153 samples)

Country	Number of sites	Submitted samples	CDI testing at sites and CDI cases determined at the ECL				Undiagnosed cases			
			Samples tested for CDI at sites (% of all submitted samples)		CDI cases (% of all samples with ECL results)		Cases not tested at sites (% of all positive cases)		Cases not tested at sites because of absence of clinical suspicion^a^ (% of all positive cases)	
	n	n	n	%	n	%	n	%	n	%
Whole healthcare system										
Belgium	1	37	18	NA	0	NA	0	NA	NA	NA
France	23	212	161	75.9	3	1.4	1	NA	1	NA
Greece	4	27	18	NA	1	NA	0	NA	0	NA
Ireland	1	21	21	NA	1	NA	0	NA	0	NA
Italy	20	222	134	60.4	15	6.9	0	NA	0	NA
Netherlands	6	196	39	19.9	2	1.0	0	NA	0	NA
Poland	12	115	87	75.7	12	10.6	1	NA	0	NA
Romania	7	159	74	46.5	25	15.8	6	NA	0	NA
Slovakia	2	23	11	NA	2	NA	0	NA	0	NA
Spain	16	666	309	46.4	13	2.0	3	NA	1	NA
Sweden	3	228	83	36.4	5	2.2	1	NA	1	NA
UK	23	1,247	737	59.1	24	2.0	12	NA	8	NA
Total	118	3,153	1,692	53.2^b^	103	2.9^c^	24	23.3	11	10.7
Hospital										
Belgium	1	34	18	NA	0	NA	NA	NA	NA	NA
France	23	187	149	79.7	3	1.6	1	NA	1	NA
Greece	4	24	18	NA	1	NA	0	NA	0	NA
Ireland	1	16	16	NA	1	NA	0	NA	0	NA
Italy	20	157	121	77.1	14	9.2	0	NA	0	NA
Netherlands	6	56	30	53.6	2	3.6	0	NA	0	NA
Poland	12	106	82	77.4	12	11.5	1	NA	0	NA
Romania	7	155	72	46.5	25	16.2	6	NA	0	NA
Slovakia	2	20	10	NA	2	NA	0	NA	0	NA
Spain	16	247	166	67.2	9	3.7	1	NA	0	NA
Sweden	3	83	54	65.1	5	6.1	1	NA	1	NA
UK	23	587	513	87.4	10	1.7	3	NA	1	NA
Total	118	1,672	1,249	75.5^b^	84	4.4^c^	13	15.5	3	3.6
Community										
Belgium	1	3	0	NA	0	NA	NA	NA	NA	NA
France	23	25	12	NA	0	NA	NA	NA	NA	NA
Greece	4	3	0	NA	0	NA	NA	NA	NA	NA
Ireland	1	5	5	NA	0	NA	NA	NA	NA	NA
Italy	20	65	13	20.0	1	1.6	0	NA	NA	NA
Netherlands	6	140	9	6.4	0	0.0	NA	NA	NA	NA
Poland	12	9	5	NA	0	NA	NA	NA	NA	NA
Romania	7	4	2	NA	0	NA	NA	NA	NA	NA
Slovakia	2	3	1	NA	0	NA	NA	NA	NA	NA
Spain	16	419	143	34.1	4	1.0	2	NA	1	NA
Sweden	3	145	29	20.0	0	0.0	NA	NA	NA	NA
UK	23	660	224	33.9	14	2.2	9	NA	8	NA
Total	118	1,481	443	29.9^b^	19	1.3^c^	11	NA	9	NA

CDI: *Clostridioides difficile* infection; ECL: European coordinating laboratory; NA: not applicable (small sample size); UK: United Kingdom.

Cases were defined as patients with samples positive for CDI by cell-cytotoxin neutralisation assay (CCNA) at the ECL.

^a^ Underlying reasons for not testing cases at participating sites, as obtained from retrospective medical records, are patients with another sample tested within 2 days of the original submitted sample and paediatric patients (< 5 years of age); no cases between the age of 5 and 17 years were identified at the ECL.

^b^ Percentage of submitted samples tested at participating sites across Europe per day.

^c^ Percentage of submitted samples positive at the ECL across participating sites in Europe per day.

Of the three truly missed hospitalised adult cases, two were not treated for CDI and were re-admitted to hospital twice and died within 4 months; one was never tested for CDI in the 6-month period following the sample, and the other was diagnosed with CDI 40 days after the original sample. All three undiagnosed hospitalised adult CDI cases were older than 65 years of age and had a history of antibiotic use or a high Charlson index (Supplementary Table S1: Metadata of undiagnosed CDI cases and Supplementary Table S2: Features, previous exposure, and co-morbidities of undiagnosed adult CDI cases). Most (8/9) of the undiagnosed adult CDI cases in the community were less than 65 years old, and five of these eight reported a history of either antibiotic use, CDI or contact with a healthcare facility. All nine undiagnosed adult cases in the community were not treated for CDI, eight were never tested for CDI in the 6-month period following the original sample, and one obtained a delayed CDI diagnosis (25 days after the original sample) and required three hospital admissions (Supplementary Tables S1 and S2).

### PCR ribotyping of *Clostridioides difficile* isolates

**Figure 2:**
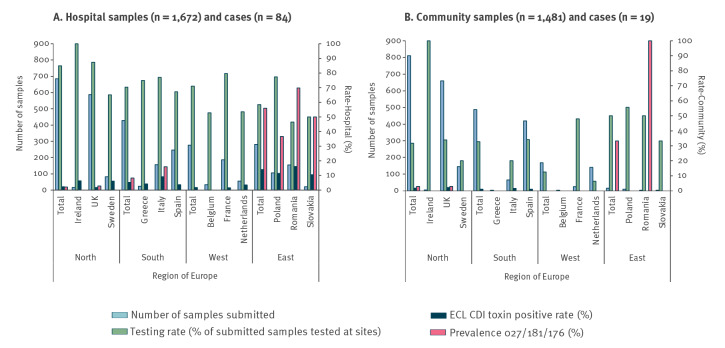
Testing rate, prevalence of related ribotypes within toxinotype IIIb (027, 181 and 176) and *Clostridioides difficile* positivity rate in diarrhoeal faecal samples A. hospital and B. community locations across countries, by European region, July–November 2018 (n = 3,153)

A total of 67 different ribotypes were identified among 198 *C. difficile* isolates from hospitals. The five most common ribotypes from hospitals were 027 (11%; n = 21), 181 (12%; n = 24), 014 (8%; n = 15), 010 (5%; n = 10), and 002 (5%; n = 9). Two ribotypes (027 and 181) are closely related, produce one of the variant toxin forms and belong to toxinotype IIIb. The highest prevalence of all toxinotype IIIb isolates (ribotypes 027,181 and 176) was seen in eastern Europe (55.9% (43/77) of all isolates in this region (country range: 36.7–69.8) which also has the lowest testing rate in a hospital setting (correlation regional testing rate vs prevalence of toxinotype IIIb r = −0.81, Figure 2). A total of 41 different ribotypes were identified among 82 *C. difficile* isolates from the community, including 181 (two isolates, 2% prevalence), with the most prevalent being 078 (9%), 039 (9%), 001 (6%), 020 (6%), 009 (5%), and 010 (5%). There were 26 ribotypes found in both hospital and community samples (totalling 193 isolates), with the most prevalent being 181 (13%), 014 (9%), 078 (8%), 010 (7%), and 039 (7%) (Supplementary Figure S2: Number of PCR ribotypes isolated from samples submitted).

CDI: *Clostridioides difficile* infection; ECL: European coordinating laboratory; UK: United Kingdom.

### Demographics of participants and clinical parameters

Retrospective follow-up data were obtained for a total of 615 participants (Table 2, Supplementary Table S3: Location of the participants), including 94 CDI cases (n = 76 in hospitals, n = 18 in the community), 34 CD (n = 18 in hospitals, n = 16 in the community), 43 SIMOApos (n = 35 in hospitals, n = 8 in the community), and 444 controls (n = 301 in hospitals, n = 143 in the community). There were similar proportions of males and females included in all categories (Table 2), with slightly fewer female cases in hospitals than in the community (53% vs 67%, p = 0.29). The median age of hospitalised cases was 74 years (IQR: 51–81), which was significantly higher than those in the community (59 years (IQR 26–71), p = 0.018). Cases were significantly older than those positive for the pathogen alone (CDI cases, median: 72 years (IQR: 51–81) vs CD, median: 47 years (IQR: 4–72), p = 0.013), and compared with controls (median: 55 years (IQR: 21–72), p < 0.0001). Patients positive for the pathogen alone were not significantly older than controls (CD, median: 47 years (IQR: 4–72); vs controls, median: 55 years (IQR: 21–72), p = 0.86).

**Table 2:** Demographics of participants with clinical data in the outcome groups for *Clostridioides difficile* infections and control group, by healthcare settings, Europe, July-November 2018 (n = 615)

Participant groups	Cases^a^		CD^b^		SIMOApos^c^		Controls		Total	
	n	%	n	%	n	%	n	%	n	%
Whole healthcare										
Total Europe	94	100.0	34	100.0	43	100.0	444	100.0	615	100.0
Belgium	0	NC	2	NC	1	NC	13	NC	16	NC
France	3		2		5		24		34	
Greece	1		0		1		4		6	
Ireland	1		0		0		3		4	
Italy	13		1		8		43		65	
Netherlands	2		1		0		9		12	
Poland	12		2		8		38		60	
Romania	18		3		5		57		83	
Slovakia	2		0		2		8		12	
Spain	13		11		5		93		122	
Sweden	5		1		0		15		21	
UK	24		11		8		137		180	
Gender										
Female	51	55.4	16	NA	18	NA	203	45.7	288	47.0
Male	41	44.6	17	NA	25	NA	241	54.3	324	53.0
Unknown	2	NC	1	NC	0	NC	0	NC	3	NC
Age (years)										
Median (IQR)	72 (51–81)		47 (4–72)		70 (52–80)		55 (21–72)		58 (28–75)	
< 18	11	11.7	13	NA	4	NA	95	21.4	123	20.0
18–49	9	9.6	4	NA	6	NA	100	22.5	119	19.3
50–64	15	15.9	4	NA	8	NA	93	20.9	120	19.5
65–84	47	50.0	11	NA	21	NA	114	25.7	193	31.4
≥ 85	12	12.8	2	NA	4	NA	42	9.5	60	9.8
Hospital										
Total Europe	76	100.0	18	100.0	35	100.0	301	100.0	430	100.0
Belgium	0	NC	1	NC	1	NC	12	NC	14	NC
France	3		2		5		24		34	
Greece	1		0		1		4		6	
Ireland	1		0		0		2		3	
Italy	13		1		5		38		57	
Netherlands	2		0		0		5		7	
Poland	12		2		8		36		58	
Romania	18		3		4		56		81	
Slovakia	2		0		2		8		4	
Spain	9		3		2		37		51	
Sweden	5		1		0		11		17	
UK	10		5		7		68		90	
Gender										
Female	39	52.7	8	NA	14	NA	139	46.2	200	46.8
Male	35	47.3	9	NA	21	NA	162	53.8	227	53.2
Unknown	2	NC	1	NC	0	NC	0	NC	3	NC
Age (years)										
Median (IQR)	74 (51–81)		64 (43–80)		70 (53–78)		59 (28–77)		62 (34–79)	
< 18	9	11.8	4	NA	4	NA	59	19.6	76	17.7
18–49	5	6.6	1	NA	4	NA	58	19.3	68	15.8
50–64	9	11.8	4	NA	7	NA	61	20.3	81	18.8
65–84	41	54.0	8	NA	17	NA	87	28.9	153	35.6
≥ 85	12	15.8	1	NA	3	NA	36	11.9	52	12.1
Community										
Total Europe	18	100.0	16	100.0	8	100.0	143	100.0	185	100.0
Belgium	0	NC	1	NC	0	NC	1	NC	2	NC
France	0		0		0		0		0	
Greece	0		0		0		0		0	
Ireland	0		0		0		1		1	
Italy	0		0		3		5		8	
Netherlands	0		1		0		4		5	
Poland	0		0		0		2		2	
Romania	0		0		1		1		2	
Slovakia	0		0		0		0		0	
Spain	4		8		3		56		71	
Sweden	0		0		0		4		4	
UK	14		6		1		69		90	
Gender										
Female	12	NA	8	NA	4	NA	64	44.8	88	47.6
Male	6	NA	8	NA	4	NA	79	55.2	97	52.4
Unknown	0	NC	0	NC	0	NC	0	NC	0	NC
Age (years)										
Median (IQR)	59 (26–71)		16 (1–50)		74 (52–82)		44 (18–63)		46 (17–66)	
< 18	2	NA	9	NA	0	NA	36	25.1	47	25.4
18–49	4	NA	3	NA	2	NA	42	29.4	51	27.6
50–64	6	NA	0	NA	1	NA	32	22.4	39	21.1
65–84	6	NA	3	NA	4	NA	27	18.9	40	21.6
≥ 85	0	NA	1	NA	1	NA	6	4.2	8	4.3

CCNA: cell-cytotoxin neutralisation assay; CTC: cell cytotoxigenic culture; IQR: interquartile range; NA: not applicable (small sample size); NC: not computed; SIMOA: ultra-sensitive toxin single molecule array; UK: United Kingdom.

^a^ Cases were the participant group that was CCNA-positive (presence of *C. difficile* toxin using reference test).

^b^ CD was the term chosen to describe the participant group that was CTC-positive/CCNA-negative/SIMOA-negative (presence of *C. difficile* without toxin).

^c^ SIMOApos was the term chosen to describe the participant group that was SIMOA-positive/CCNA-negative (presence of *C. difficile* toxin using novel test).

Hospitalised cases had a significantly higher mean white blood cell count than that of controls (15.2 vs 10.4x10^9^/L, p = 0.01. Normal range: 4.5–11.0x10^9^/L) (Table 3). For all patients, there was no significant difference in the median number of days of diarrhoea before the sample being taken compared with controls (Table 3). However, community cases had significantly more days of diarrhoea before a sample was submitted compared with those cases in the hospital setting (median: 10 days vs 2 days, p = 0.01). Among all patients in hospitals, 6/64 cases, 2/18 CD, and 1/28 SIMOApos had prior CDI in the 8 weeks preceding sample acquisition (Table 3). None of the cases or CD in the community with reported data had prior CDI. However, one of the five SIMOApos participants in the community with reported data was a recurrent case (Table 3). The proportion of hospital cases re-admitted to hospital within 30 days was similar to that of controls (15.7% (11/70) vs 10.3% (29/282), p = 1.00). The proportion of community cases admitted to hospital within 30 days of the sample being taken was not significantly different to that of controls, (2/17 vs 14/126, p = 1.00). However, a higher proportion of community SIMOApos were admitted to hospital within 30 days compared with controls (3/7 vs 14/126, p = 0.04). The 30-day mortality rate was significantly higher for hospital cases compared with controls (18.6% (13/70) vs 8.6% (23/269), p = 0.01, Table 3).

**Table 3:** Clinical parameters of participants, comparing outcome groups for *Clostridioides difficile* infections and control group, by healthcare settings, Europe, July–November 2018 (n = 615)

Parameters		White blood cell count (per 10^9^/L)				Days of diarrhoea before sampling				Recurrent (previous) *C. difficile* infection		Hospital admission within 30 days			30-day mortality rate		
**Participant groups**	**Healthcare settings**	**n**	**Mean **	**SD**	**p**	**n**	**Median **	**IQR**	**p**	**n/N**	**%**	**n/N**	**%**	**p**	**n/N**	**%**	**p**
Cases^a^	Total	70	14.8	13.6	**0.01**	68	3	1–7	0.75	6/82	7.3	13/87	14.9	0.24	14/88	15.9	**0.01**
	Hospital	65	15.2	14.0	**0.01**	59	2	1–5	0.61	6/64	9.4	11/70	15.7	0.20	13/70	18.6	**0.01**
	Community	5	9.5	4.8	0.86	9	10	6–14	0.93	0/18	NA	2/17	NA	1.00	1/18	NA	0.23
CD^b^	Total	20	10.7	5.2	0.70	18	2	1–15	0.97	2/33	NA	3/32	NA	1.00	3/32	NA	0.44
	Hospital	15	10.9	5.8	0.78	12	1	1–10	0.43	2/18	NA	2/18	NA	1.00	3/17	NA	0.19
	Community	5	10.4	3.3	0.54	6	14	7–15	0.64	0/15	NA	1/14	NA	1.00	0/15	NA	1.00
SIMOApos^c^	Total	33	13.6	10.3	0.08	26	3	1–7	0.96	2/33	NA	6/41	NA	0.43	3/40	NA	0.73
	Hospital	30	13.8	10.8	0.11	24	2	1–7	0.81	1/28	NA	3/34	NA	1.00	2/33	NA	1.00
	Community	3	12.1	3.5	0.28	2	4	4–4	0.54	1/5	NA	3/7	NA	**0.04**	1/7	NA	0.10
Controls	Total	291	10.3	8.0	Ref	258	3	1–7	Ref	NR	NR	43/408	10.5	Ref	24/401	6.0	Ref
	Hospital	259	10.4	7.9	Ref	193	2	1–4	Ref	NR	NR	29/282	10.3	Ref	23/269	8.6	Ref
	Community	32	9.0	9.2	Ref	65	8	5–20	Ref	NR	NR	14/126	11.1	Ref	1/132	0.8	Ref

CCNA: cell-cytotoxin neutralisation assay; CTC: cell cytotoxigenic culture; IQR: interquartile range of median; n: number of participants with indicated parameters; N: total number of participants in analysis (with data on the indicated parameters available from medical records); NA: not applicable (small sample size); NR: not relevant as one of the criteria for the control groups was to not have had previous *C. difficile* infection; Ref: reference for the calculation of p; SD: standard deviation of mean; SIMOA: ultra-sensitive toxin single molecule array.

^a^ Cases were the participant group that was CCNA-positive (presence of *C. difficile* toxin using reference test).

^b^ CD was the term chosen to describe the participant group that was CTC-positive/CCNA-negative/SIMOA-negative (presence of *C. difficile* without toxin).

^c^ SIMOApos was the term chosen to describe the participant group that was SIMOA-positive/CCNA-negative (presence of *C. difficile* toxin using novel test).

The p value for each group was compared to controls; significance (p ≤ 0.05) is denoted with bold text. The full number of participants in each group is shown in Table 2.

### Previous exposure to healthcare setting or antibiotics, co-morbidities

**Figure 3:**
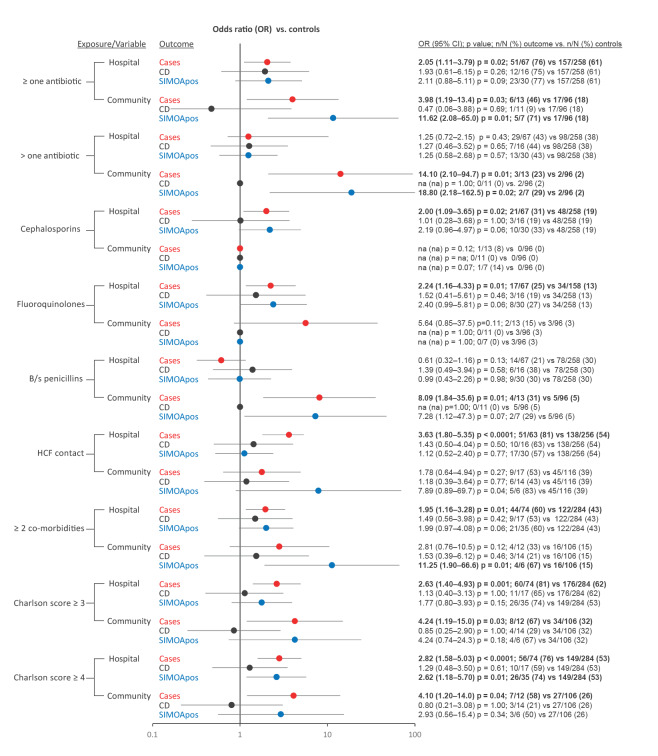
Forest plot of odds ratio for previous exposure and co-morbidities, comparing outcome groups for *Clostridioides difficile* infections and control groups, by healthcare settings, Europe, 2018 (n = 615)

CCNA: cell-cytotoxin neutralisation assay; CTC: cell cytotoxigenic culture; B/s: broad spectrum; CI: confidence intervals; HCF: Healthcare facility; OR: odds ratio. n: number of participants exposed to indicated variable. N: total number of participants in analysis (with data on the indicated variables available from medical records); NC: not computed because of a value of 0 in one of the groups; SIMOA: ultra-sensitive toxin single molecule array.

^a^ Cases were the participant group that was CCNA-positive (presence of *C. difficile* toxin using reference test).

^b^ CD was the term chosen to describe the participant group that was CTC-positive/CCNA-negative/SIMOA-negative (presence of *C. difficile* without toxin).

^c^ SIMOApos was the term chosen to describe the participant group that was SIMOA-positive/CCNA-negative (presence of *C. difficile* toxin using novel test).

Cases in hospital and community settings were significantly more likely to have been prescribed antibiotics in the preceding 12 weeks of the sample being taken (p = 0.02, p = 0.03, respectively, Figure 3), while cases and the SIMOApos group in the community were more likely to have taken more than one antibiotic compared with controls (p = 0.01, p = 0.02, respectively, Figure 3). Previous exposure to cephalosporins and fluoroquinolones was reported in a higher proportion of hospital cases compared with controls (31.3% vs 18.6%, OR: 2.00 (1.09–3.65), p = 0.02, and 25.4% vs 13.2%, OR: 2.24 (1.16–4.33), p = 0.01, respectively), while exposure to broad-spectrum penicillins was significantly more frequent in community cases compared with controls (30.8% vs 5.2%, OR: 8.09 (1.84–35.60), p = 0.01). Previous exposure to a healthcare facility was reported in a significantly higher proportions of hospital cases compared with controls (81.0% vs 53.9%, OR = 3.63 (1.80–5.35), p < 0.0001). Hospitalised cases were associated with a higher number of co-morbidities (OR = 1.95 (1.16–3.28), p = 0.01), and a significant association was also identified in the community SIMOApos group (p = 0.01). An elevated Charlson index score was associated with cases and the SIMOApos group in the hospital (score ≥ 3 for hospital cases, OR: 2.63 (1.40–4.93), p = 0.001; score ≥ 4 for hospital SIMOApos, OR: 2.62 (1.18–5.70), p = 0.01). Similarly, an elevated Charlson index score was found associated with cases in the community (score ≥ 3, OR: 4.24 (1.19–15.0), p = 0.03). In both healthcare settings, there were no significant differences in the percentage of patients positive for the pathogen alone with a reported exposure or co-morbidities compared with controls (CD, Figure 3).

Categorical variables with a statistically significant OR between outcome and controls groups in at least one of the healthcare settings are shown, among the following variables: number and type of antibiotics used in the 12 weeks preceding the sample, contact with a healthcare facility in the previous 6 months, contact with a case or infant in the previous 3 months to the sample being taken, number of co-morbidities and Charlson index score (Supplementary Table S4: Categorical variables (previous exposure) of cases and CD participants groups and Supplementary Table S5: Categorical variables (number and type of co-morbidities) of cases and CD participants group; Supplementary Table S6: Categorical variables (previous exposure) of SIMOApos participants group and Supplementary Table S7: Categorical variables (number and type of co-morbidities) of SIMOApos participants group). Significant risk factors (p values < 0.05 and 95% CI that do not include 1.0) are highlighted in bold.

## Discussion

Our study examines the testing and positivity rate for CDI in diarrhoeal samples collected in hospital and community settings across 12 European countries between July to November 2018, including description of undiagnosed cases and of predisposing risk factors for CDI. One third of diarrhoeal samples submitted for diagnostic investigations in the community setting were tested for CDI, compared with three quarters for hospitalised patients, supporting findings of lower clinical suspicion for this infection within the community [8,12,14]. The overall positivity rate of CDI in submitted diarrhoeal samples, as determined by the reference test CCNA result performed at the ECL, was lower in community samples than that seen in the hospital samples.

The proportion of undiagnosed hospitalised CDI cases in this study is slightly lower than the proportion reported in the 2012–13 EUCLID study (16% vs 23%) [15]. The retrospective medical records allowed us to determine that the proportion of true undiagnosed adult hospitalised CDI cases in the COMBACTE-CDI study was minimal (4%). However, this study reports that 47% of the adult CDI cases who submitted a diarrhoeal sample in a community setting were never tested for CDI. Assuming that the measured daily rate of under-diagnosis is constant, this would lead to ca 1,700 undiagnosed adult cases in the community per year across the 118 participating sites, compared with ca 550 undiagnosed adult cases in the hospital. As we recruited one site per 3 million population in each country, the true number of undiagnosed infections in the community may far exceed this. As previously described [15] and considering there are around 8,000 hospitals across Europe, this would equate to ca 37,000 undiagnosed hospitalised cases (meaning that there could be ca 19% more CDI cases than reported in ECDC 2016–17 point prevalence surveys [1]), with a number three times higher (ca 111,000) in the community across Europe per year. In addition, there was evidence of previous antibiotic use or contact with a healthcare facility in the 6 months preceding the sample in the majority of undetected adult cases younger than 65 years of age in the community; this highlights that *C. difficile* testing was not requested in the community, even when the patient had known predisposing risk factors for the infection. For hospital samples, the observation of the relationship between testing rate and prevalence of 027 has been previously demonstrated [15]; our findings in this study expand this previous observation to other closely related ribotypes (181 and 176). The most prevalent ribotype identified in this study present in both hospital and community settings was 181; it should be noted that 181 has been associated with an outbreak in a community rehabilitation centre [29].

This study showed that different classes of antibiotics increased the odds of developing CDI in different settings; cephalosporins or fluoroquinolones in hospitalised patients, and broad-spectrum penicillins in the community. This may reflect the different classes of antibiotics that are used in these two different healthcare settings. Indeed, half of community CDI cases in this study had no previous history of antibiotic use, as previously described [13]. Interestingly, community cases had five times more days of diarrhoea before a sample was submitted compared with those in the hospital setting, consistent with previous observations from the Netherlands [10]. This confirms that a longer time elapsed before seeking medical care and/or a reduced inclination to test in the community. Significant risk factors were observed in both settings for patients with *C. difficile* toxin detected only with the novel sensitive SIMOA technology, suggesting that the SIMOA test has the potential to diagnose true CDI [24]. For patients in both settings, increasing numbers of co-morbidities were a risk factor for toxin-positive CDI, while previous contact with a healthcare facility was only a significant risk factor for those cases within the hospital. We observed a higher white blood cell count and 30-day mortality rate in hospitalised patients with CDI, but not for those with only the pathogen detected (no free toxin (CD)), consistent with previous observations [30]. Indeed, no risk factors were identified in this study for the presence of *C. difficile* alone (CD), confirming that these are unlikely to be true CDI [22,23,30].

There are some limitations to the study. It is worth noting that the COMBACTE-CDI project focused on the recruitment of a comparable and balanced number of sites across all four regions of Europe (ca 20–40 sites per region, with three countries per region). The study therefore did not include Germany because of its high population and the number of sites that would have been needed to be recruited (ca 30 sites; one site per 3 million population), which would have resulted in overrepresentation of the western region, as can be seen in data from the 2012–13 EUCLID study [15]. Although we included all diarrhoeal samples that were sent to a laboratory, the incidence of CDI in our study could be underestimated if diarrhoeal samples of patients with CDI were not sent to a laboratory and the disease had a mild and self-limiting course. We were also unable to determine the precise origin of infection in hospitalised patients, i.e. healthcare-associated vs community-associated CDI. Although children under the age of 2 years were included as part of the submitted samples; these were removed from the follow-up analysis of undiagnosed adult cases of CDI, and a representative sample of each age group was included in the case–control study. The number of community patients included in this study was small; however, our data still identified significant predisposing risk factors for community CDI.

## Conclusions

Findings from this study highlight the burden that undiagnosed CDI places on healthcare systems, whereby lack of clinical suspicion and testing in adults who present with diarrhoea in the hospital or community setting was found to ultimately lead to further hospital admissions. Increased awareness by both patients and healthcare professionals of CDI in the community, in adults with either known predisposing risk factors or in those with prolonged days of diarrhoea, is required to reduce transmission rates and potential further healthcare burden.

## Supplementary Data

470000 Supplement

## References

[1] Suetens C Latour K Kärki T Ricchizzi E Kinross P Moro ML The Healthcare-Associated Infections Prevalence Study Group . Prevalence of healthcare-associated infections, estimated incidence and composite antimicrobial resistance index in acute care hospitals and long-term care facilities: results from two European point prevalence surveys, 2016 to 2017. Euro Surveill. 2018;23(46):1800516. 10.2807/1560-7917.ES.2018.23.46.1800516 30458912 PMC6247459

[2] Kwon JH Olsen MA Dubberke ER . The morbidity, mortality, and costs associated with Clostridium difficile infection. Infect Dis Clin North Am. 2015;29(1):123-34. 10.1016/j.idc.2014.11.003 25677706

[3] Davies K Lawrence J Berry C Davis G Yu H Cai B Risk factors for primary Clostridium difficile infection; results from the observational study of risk factors for Clostridium difficile infection in hospitalized patients with infective diarrhea (ORCHID). Front Public Health. 2020;8:293. 10.3389/fpubh.2020.00293 32766196 PMC7379483

[4] Bartlett JG Gerding DN . Clinical recognition and diagnosis of Clostridium difficile infection. Clin Infect Dis. 2008;46(s1) Suppl 1;S12-8. 10.1086/521863 18177217

[5] Rodríguez-Pardo D Almirante B Bartolomé RM Pomar V Mirelis B Navarro F Barcelona Clostridium difficile Study Group . Epidemiology of Clostridium difficile infection and risk factors for unfavorable clinical outcomes: results of a hospital-based study in Barcelona, Spain. J Clin Microbiol. 2013;51(5):1465-73. 10.1128/JCM.03352-12 23447638 PMC3647904

[6] Abou Chakra CN Pepin J Sirard S Valiquette L . Risk factors for recurrence, complications and mortality in Clostridium difficile infection: a systematic review. PLoS One. 2014;9(6):e98400. 10.1371/journal.pone.0098400 24897375 PMC4045753

[7] Marra F Ng K . Controversies around epidemiology, diagnosis and treatments of Clostridium difficile infection. Drugs. 2015;75(10):1095-118. 10.1007/s40265-015-0422-x 26113167

[8] Ofori E Ramai D Dhawan M Mustafa F Gasperino J Reddy M . Community-acquired Clostridium difficile: epidemiology, ribotype, risk factors, hospital and intensive care unit outcomes, and current and emerging therapies. J Hosp Infect. 2018;99(4):436-42. 10.1016/j.jhin.2018.01.015 29410012

[9] Deshpande A Pasupuleti V Thota P Pant C Rolston DDK Sferra TJ Community-associated Clostridium difficile infection and antibiotics: a meta-analysis. J Antimicrob Chemother. 2013;68(9):1951-61. 10.1093/jac/dkt129 23620467

[10] Crobach MJT Notermans DW Harmanus C Sanders IMJG De Greeff SC Kuijper EJ . Community-Onset *Clostridioides Difficile* Infection in Hospitalized Patients in The Netherlands. Open Forum Infect Dis. 2019;6(12):ofz501. 10.1093/ofid/ofz501 31844637 PMC6904416

[11] Khanna S Pardi DS Aronson SL Kammer PP Orenstein R St Sauver JL The epidemiology of community-acquired Clostridium difficile infection: a population-based study. Am J Gastroenterol. 2012;107(1):89-95. 10.1038/ajg.2011.398 22108454 PMC3273904

[12] Hensgens MP Dekkers OM Demeulemeester A Buiting AGM Bloembergen P van Benthem BHB Diarrhoea in general practice: when should a Clostridium difficile infection be considered? Results of a nested case-control study. Clin Microbiol Infect. 2014;20(12):O1067-74. 10.1111/1469-0691.12758 25040463

[13] Wilcox MH Mooney L Bendall R Settle CD Fawley WN . A case-control study of community-associated Clostridium difficile infection. J Antimicrob Chemother. 2008;62(2):388-96. 10.1093/jac/dkn163 18434341

[14] Barbut F Day N Bouée S Youssouf A Grandvoinnet L Lalande V Toxigenic Clostridium difficile carriage in general practice: results of a laboratory-based cohort study. Clin Microbiol Infect. 2019;25(5):588-94. 10.1016/j.cmi.2018.12.024 30616013

[15] Davies KA Longshaw CM Davis GL Bouza E Barbut F Barna Z Underdiagnosis of Clostridium difficile across Europe: the European, multicentre, prospective, biannual, point-prevalence study of Clostridium difficile infection in hospitalised patients with diarrhoea (EUCLID). Lancet Infect Dis. 2014;14(12):1208-19. 10.1016/S1473-3099(14)70991-0 25455988

[16] Dubberke ER Olsen MA . Burden of Clostridium difficile on the healthcare system. Clin Infect Dis. 2012;55(Suppl 2):S88-92. 10.1093/cid/cis335 22752870 PMC3388018

[17] Freeman J Bauer MP Baines SD Corver J Fawley WN Goorhuis B The changing epidemiology of Clostridium difficile infections. Clin Microbiol Rev. 2010;23(3):529-49. 10.1128/CMR.00082-09 20610822 PMC2901659

[18] Davies KA Ashwin H Longshaw CM Burns DA Davis GL Wilcox MH EUCLID study group . Diversity of Clostridium difficile PCR ribotypes in Europe: results from the European, multicentre, prospective, biannual, point-prevalence study of Clostridium difficile infection in hospitalised patients with diarrhoea (EUCLID), 2012 and 2013. Euro Surveill. 2016;21(29):30294. 10.2807/1560-7917.ES.2016.21.29.30294 27470194

[19] He M Miyajima F Roberts P Ellison L Pickard DJ Martin MJ Emergence and global spread of epidemic healthcare-associated Clostridium difficile. Nat Genet. 2013;45(1):109-13. 10.1038/ng.2478 23222960 PMC3605770

[20] Goorhuis A Bakker D Corver J Debast SB Harmanus C Notermans DW Emergence of Clostridium difficile infection due to a new hypervirulent strain, polymerase chain reaction ribotype 078. Clin Infect Dis. 2008;47(9):1162-70. 10.1086/592257 18808358

[21] Fawley WN Davies KA Morris T Parnell P Howe R Wilcox MH Clostridium difficile Ribotyping Network (CDRN) Working Group . Enhanced surveillance of Clostridium difficile infection occurring outside hospital, England, 2011 to 2013. Euro Surveill. 2016;21(29):30295. 10.2807/1560-7917.ES.2016.21.29.30295 27487436

[22] Crobach MJT Planche T Eckert C Barbut F Terveer EM Dekkers OM European Society of Clinical Microbiology and Infectious Diseases: update of the diagnostic guidance document for Clostridium difficile infection. Clin Microbiol Infect. 2016;22(Suppl 4):S63-81. 10.1016/j.cmi.2016.03.010 27460910

[23] Planche T Wilcox M . Reference assays for Clostridium difficile infection: one or two gold standards? J Clin Pathol. 2011;64(1):1-5. 10.1136/jcp.2010.080135 21118850

[24] Banz A Lantz A Riou B Foussadier A Miller M Davies K Sensitivity of single-molecule array assays for detection of Clostridium difficile toxins in comparison to conventional laboratory testing algorithms. J Clin Microbiol. 2018;56(8):e00452-18. 10.1128/JCM.00452-18 29898996 PMC6062787

[25] Freeman J Wilcox MH . The effects of storage conditions on viability of Clostridium difficile vegetative cells and spores and toxin activity in human faeces. J Clin Pathol. 2003;56(2):126-8. 10.1136/jcp.56.2.126 12560391 PMC1769877

[26] Bouza E Peláez T Alonso R Catalán P Muñoz P Créixems MR . "Second-look" cytotoxicity: an evaluation of culture plus cytotoxin assay of Clostridium difficile isolates in the laboratory diagnosis of CDAD. J Hosp Infect. 2001;48(3):233-7. 10.1053/jhin.2001.1000 11439012

[27] Fawley WN Knetsch CW MacCannell DR Harmanus C Du T Mulvey MR Development and validation of an internationally-standardized, high-resolution capillary gel-based electrophoresis PCR-ribotyping protocol for Clostridium difficile. PLoS One. 2015;10(2):e0118150. 10.1371/journal.pone.0118150 25679978 PMC4332677

[28] Stubbs SL Brazier JS O’Neill GL Duerden BI . PCR targeted to the 16S-23S rRNA gene intergenic spacer region of Clostridium difficile and construction of a library consisting of 116 different PCR ribotypes. J Clin Microbiol. 1999;37(2):461-3. 10.1128/JCM.37.2.461-463.1999 9889244 PMC84342

[29] Kachrimanidou M Baktash A Metallidis S Tsachouridou Ο Netsika F Dimoglou D An outbreak of Clostridioides difficile infections due to a 027-like PCR ribotype 181 in a rehabilitation centre: Epidemiological and microbiological characteristics. Anaerobe. 2020;65:102252. 10.1016/j.anaerobe.2020.102252 32781108

[30] Planche TD Davies KA Coen PG Finney JM Monahan IM Morris KA Differences in outcome according to Clostridium difficile testing method: a prospective multicentre diagnostic validation study of C difficile infection. Lancet Infect Dis. 2013;13(11):936-45. 10.1016/S1473-3099(13)70200-7 24007915 PMC3822406

