# Supplementary material for: A point-prevalence study on community and inpatient Clostridioides difficile infections (CDI): results from Combatting Bacterial Resistance in Europe CDI (COMBACTE-CDI), July to November 2018
Source: Euro Surveill. 2022 Jun 30;27(26):2100704. doi: 10.2807/1560-7917.ES.2022.27.26.2100704 (PMC9248264; doi:10.2807/1560-7917.ES.2022.27.26.2100704)
Supplement: Supplement [file 21-00704_DAVIES_Supplement.pdf]

Supplementary material

This supplementary material is hosted by *Eurosurveillance* as supporting information alongside the article "A point-prevalence study on community and inpatient *Clostridioides difficile* infections (CDI): results from Combatting Bacterial Resistance in Europe CDI (COMBACTE-CDI), July to November 2018", on behalf of the authors who remain responsible for the accuracy and appropriateness of the content. The same standards for ethics, copyright, attributions and permissions as for the article apply. Supplements are not edited by *Eurosurveillance* and the journal is not responsible for the maintenance of any links or email addresses provided therein.

Content:

Supplementary Figure S1. Organisms detected by the BioFire FilmArray GI Panel assay.

Supplementary Table S1. Metadata of undiagnosed CDI cases.

Supplementary Table S2. Features, previous exposure, and co-morbidities of undiagnosed adult CDI cases.

Supplementary Figure S2. Number of PCR ribotypes isolated from samples submitted.

Supplementary Table S3. Location of the participants.

Supplementary Table S4. Categorical variables (previous exposure) of cases and CD participants groups.

Supplementary Table S5. Categorical variables (number and type of co-morbidities) of cases and CD participants groups.

Supplementary Table S6. Categorical variables (exposure) of SIMOApos participants group.

Supplementary Table S7. Categorical variables (number and type of co-morbidities) of SIMOApos participants group.

Supplementary Figure S1. Organisms detected by the BioFire FilmArray GI Panel assay.

Samples were positive or negative for *C. difficile* toxin (Cell-cytotoxin neutralisation assay; CCNA).

None= None detected. ADENOVLDS=Adenovirus. ASTROVLDS=Astrovirus. CAMPLYDS=Campylobacter. CRYPOSLDS=Cryptosporidium. CYCCAYLDS= Cyclospora cayetanensis. EAECLDS=E. coli Enteroaggreagative (EAEC). EIECLDS= Shigella/Enteroinvasive E. coli (EIEC). ENTHISLDS=Entamoeba histolytica. EPECLDS=E. coli Enteropathogenic (EPEC). ETECLDS=E. coli Enterotoxigenic (ETEC). GIRLAMLDS=Giardia lamblia. NOVOVLDS=Norovirus. O157LDS=E. coli O157. PLESHILDS=Plesiomonas Shigelloides. ROTAVLDS=Rotavirus. SALMLALDS=Salmonella. SAPOVLDS=Sapovirus. STECLDS=E. coli Shiga - Like Toxin - Producing (STEC). VIBCHLLDS=Vibrio Cholerae. VICRIOLDS=Vibrio. YERENTLDS=Yersinia Enterocolitica.

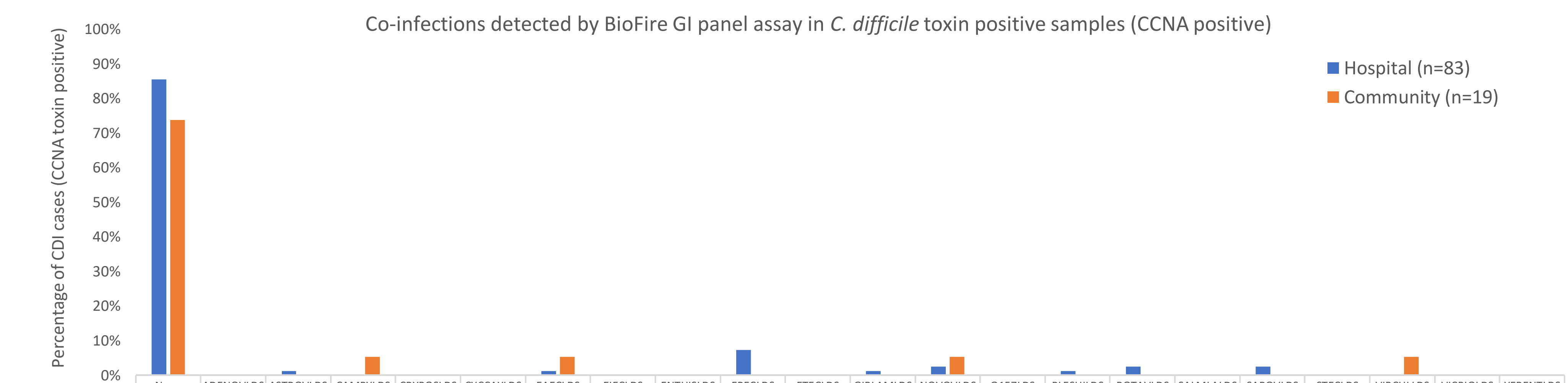

	None	ADENOVLDS	ASTROVLDS	CAMPYLDS	CRYPOSLDS	CYCCAYLDS	EAECLDS	EIECLDS	ENTHISLDS	EPECLDS	ETECLDS	GIRLAMLDS	NOVOVLDS	O157LDS	PLESHILDS	ROTAVLDS	SALMLALDS	SAPOVLDS	STECLDS	VIBCHLLDS	VICRIOLDS	YERENTLDS
Hospital (n=83)	85.5%	0.0%	1.2%	0.0%	0.0%	0.0%	1.2%	0.0%	0.0%	7.2%	0.0%	1.2%	2.4%	0.0%	1.2%	2.4%	0.0%	2.4%	0.0%	0.0%	0.0%	0.0%
Community (n=19)	73.7%	0.0%	0.0%	5.3%	0.0%	0.0%	5.3%	0.0%	0.0%	0.0%	0.0%	0.0%	5.3%	0.0%	0.0%	0.0%	0.0%	0.0%	0.0%	5.3%	0.0%	0.0%

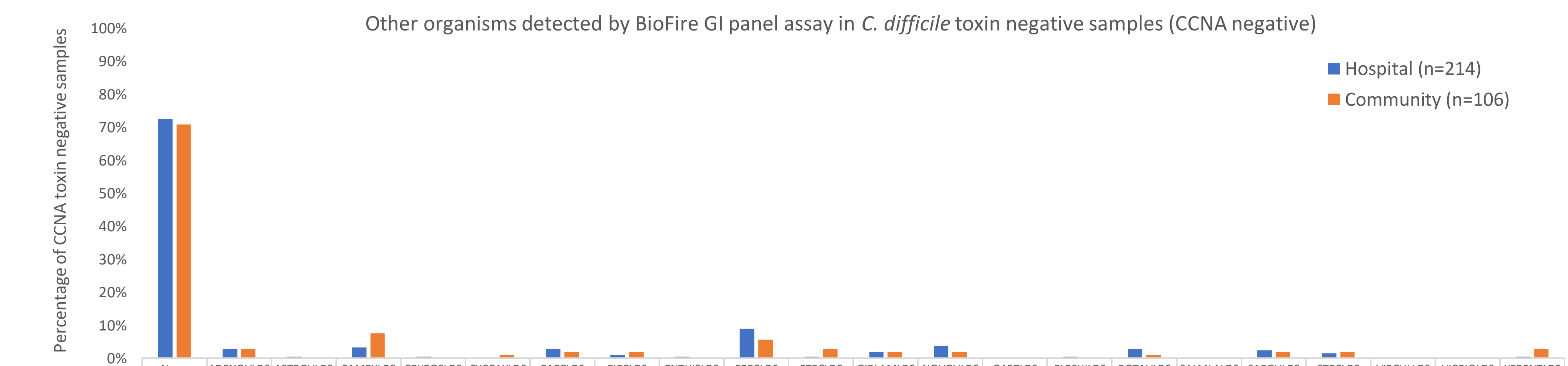

	None	ADENOVLDS	ASTROVLDS	CAMPYLDS	CRYPOSLDS	CYCCAYLDS	EAECLDS	EIECLDS	ENTHISLDS	EPECLDS	ETECLDS	GIRLAMLDS	NOVOVLDS	O157LDS	PLESHILDS	ROTAVLDS	SALMLALDS	SAPOVLDS	STECLDS	VIBCHLLDS	VICRIOLDS	YERENTLDS
Hospital (n=214)	72.4%	2.8%	0.5%	3.3%	0.5%	0.0%	2.8%	0.9%	0.5%	8.9%	0.5%	1.9%	3.7%	0.0%	0.5%	2.8%	0.0%	2.3%	1.4%	0.0%	0.0%	0.5%
Community (n=106)	70.8%	2.8%	0.0%	7.5%	0.0%	0.9%	1.9%	1.9%	0.0%	5.7%	2.8%	1.9%	1.9%	0.0%	0.0%	0.9%	0.0%	1.9%	1.9%	0.0%	0.0%	2.8%

Supplementary Table S1. Metadata of undiagnosed CDI cases.

Metadata was as reported in medical records, Europe, 2018.

CDI= *Clostridioides difficile* infection

n/a= Data not available

Missed cases #	Age groups in years	CDI test previous 6 months	Other enteropathogens positive result if tested	Diagnosis other than CDI	Outcome CDI tested following 6 months	Outcome Admitted to hospital within 30 days	Outcome Admitted to hospital 30 days - 6 months	Outcome Died	Summary finding: Reason for not being tested
**Hospital**									
#1	<5	No	none detected	Feeding difficulties	n/a	No	No	No	Paediatric
#2	<5	No	none detected	Dehydration	n/a	No	No	No	Paediatric
#3	<5	No	Rotavirus	Rotavirus infection	No	Yes (5 days) for diarrhoea and rotavirus	Yes (57, 77, 113 days) for diarrhoea and renal failure	No	Paediatric and other infection detected
#4	<5	No	Adenovirus, Metapneumovirus	Viral pneumonia	No	No	No	No	Paediatric and other infection detected
#5	<5	No	Rotavirus	Enterocolitis	n/a	No	No	No	Paediatric and other infection detected
#6	<5	No	Respiratory Enterovirus	Acute gastroenteritis	No	No	No	No	Paediatric and other infection detected
#7	65-84	No	none detected	Lymphoma, Chronic obstructive pulmonary disease	No	No	No	No	Missed case; no report of CDI testing but was treated with antibiotic for CDI
#8	65-84	Yes	Adenovirus, Norovirus, Rotavirus	Steatohepatitis non alcoholic	n/a	No	n/a	No	Another sample tested positive, not true missed case
#9	65-84	Yes	No other test done	Data not available	No	Yes (date unknown)	Yes (118, 135 days) for abdominal pain	Yes (139 days)	Missed case; recurrent CDI (positive 53 days before, but negative 30 days before sample)
#10	65-84	No	none detected	Pneumonia	Yes positive (40,80 days)	No	Yes (33, 78 days,) for pneumonia	Yes (84 days)	Missed case; lack clinical suspicion

#11	>85	Yes	none detected	Dehydration	n/a	n/a	n/a	n/a	Another sample tested positive, not true missed case
**Community**									
#1	<5	No	none detected	Prolonged diarrhoea	No	No	No	No	Paediatric
#2	<5	No	No other test done	Data not available	No	No	No	No	Paediatric
#3	18-49	No	Enterohaemorrhagic E. coli	Gastroenteritis	No	No	No	No	Missed case; CDI not suspected as other infection detected
#4	18-49	No	No other test done	Gastroenteritis	No	No	No	No	Missed case; lack clinical suspicion
#5	18-49	No	Campylobacter	Data not available	No	Data not available	Data not available	No	Missed case; CDI not suspected as other infection detected
#6	18-49	No	none detected	None	No	No	No	No	Missed case; lack clinical suspicion
#7	50-64	No	No other test done	Complications of surgical and medical care	No	No	No	No	Missed case; lack clinical suspicion
#8	50-64	No	No other test done	Data not available	No	No	No	No	Missed case; lack clinical suspicion
#9	50-64	No	No other test done	Diarrhoea	Yes positive (25, 57, 79, 125, 163 days)	Yes (25 days) for diarrhoea	Yes (58, 80 days) for CDI	No	Missed case; lack clinical suspicion
#10	50-64	Yes negative (102 days)	none detected	Data not available	Yes negative (2 days)	No	No	Yes (3 days)	Missed case; test for CDI was negative 102 days before sample
#11	65-84	No	none detected	Data not available	No	No	No	No	Missed case; lack clinical suspicion

Supplementary Table S2. Features, previous exposure, and co-morbidities of undiagnosed adult CDI cases.

Features, previous exposure, and co-morbidities, as reported in medical records, Europe, 2018.

CDI= *Clostridioides difficile* infection

n/a= Data not available

Missed cases #	Age groups in years	Days of diarrhoea before the sample	Contact HCF (6 months prior)	Contact infant (3 months prior)	Antibiotic use (12 weeks prior)	Co-morbidities	Charlson Index	White cell count 10^9/Liter	Has the patient ever had CDI?	Treated with antibiotic for CDI
**Hospital**										
#7	65-84	40	Yes	n/a	Cephalosporins, Clindamycin, Fluoroquinolones	Chronic obstructive pulmonary disease, Pneumonia, Solid tumour within last 5 years	7	21.00	Yes	Yes
#9	65-84	1	Yes	n/a	Cephalosporins	Diabetes without organ damage	6	13.20	Yes	No
#10	65-84	n/a	No	n/a	No	Congestive heart failure, Cerebrovascular disease, Diabetes without organ damage	8	8.00	No	No
**Community**										
#3	18-49	5	No	n/a	No	n/a	n/a	16.10	No	No
#4	18-49	14	No	n/a	n/a	n/a	n/a	n/a	No	No
#5	18-49	n/a	n/a	n/a	n/a	n/a	n/a	n/a	No	n/a
#6	18-49	28	Yes	Yes	Broad-spectrum Penicillins	None	0	n/a	n/a	No
#7	50-64	n/a	Yes	n/a	n/a	Other	2	n/a	No	No
#8	50-64	n/a	No	n/a	Yes: n/a	n/a	n/a	n/a	No	No
#9	50-64	6	No	n/a	No	n/a	n/a	n/a	Yes	No
#10	50-64	n/a	Yes	n/a	Broad-spectrum Penicillins, Aminoglycosides, Fluoroquinolones	Chronic kidney disease, Liver disease	5	12.90	No	No
#11	65-84	n/a	No	n/a	No	Peripheral vascular disease	5	n/a	No	No

Supplementary Figure S2. Number of PCR ribotypes isolated from samples submitted.

Numbers are shown in the Venn diagram. The five most common PCR ribotypes isolated from samples submitted across Europe are shown below the Venn diagram (left: isolated from hospital samples; middle: isolated from both hospital and community samples; right: isolated from community samples). PCR ribotypes found in both hospital and community samples are also listed.

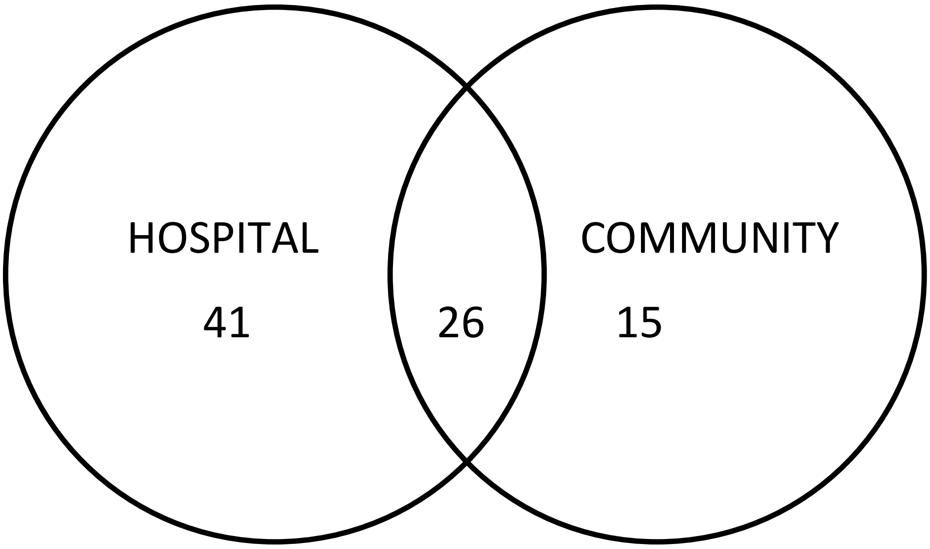

Most common

1.	181	181	078
2.	027	014	039
3.	014	078	001
4.	010	010	020
5.	002	039	009, 010

PCR ribotypes found in both hospital and community samples

181, 014, 078, 010, 039, 002, 020, 001, 003, 005, 106, 009, 015, 018, 046, 026, 035, 126, 023, 031, 013, 070, 154, 430, 815, 902.

Supplementary Table S3. Location of the participants.

Location of the participants at the time of sampling as reported in medical records.
Cases were participants group CCNA positive (presence of *C. difficile* toxin using reference test).
CD term was chosen to describe participants group CTC positive/CCNA negative/SIMOA negative (presence of *C. difficile* without toxin).
SIMOApos term was chosen to describe participants group SIMOA positive/CCNA negative (presence of *C. difficile* toxin using novel test).

CCNA: cell-cytotoxin neutralisation assay; CTC: cell cytotoxigenic culture; SIMOA: ultra-sensitive toxin single molecule array.

	**Cases**		**CD**		**SIMOApos**		**Controls**	
	**Hospital**	**Community**	**Hospital**	**Community**	**Hospital**	**Community**	**Hospital**	**Community**
**Number of patients (%)**	76 (100%)	18 (100%)	18 (100%)	16 (100%)	35 (100%)	8 (100%)	301 (100%)	143 (100%)
**Admitting speciality**								
Elderly medicine	13 (17%)	..	2 (11%)	..	3 (9%)		25 (8%)	..
Emergency medicine	11 (15%)	..	2 (11%)	..	2 (6%)		42 (14%)	..
Gastroenterology	6 (8%)	..	2 (11%)	..	3 (9%)		22 (7%)	..
Haematology/Oncology	5 (7%)	..	1 (6%)	..	4 (11%)		35 (12%)	..
Intensive care unit	1 (1%)	..	0	..	1 (3%)		17 (6%)	..
Infectious diseases	16 (21%)	..	1 (6%)	..	4 (11%)		50 (17%)	..
Surgery	6 (8%)	..	2 (11%)	..	2 (6%)		13 (4%)	
General/Acute Medicine	3 (4%)		2 (11%)		6 (17%)		65 (22%)	..
Paediatric	4 (5%)	..	1 (6%)	..	1 (3%)		22 (7%)	..
Other	10 (13%)	..	5 (28%)	..	8 (23%)		5 (2%)	..
Unknown	1 (1%)	..	0	..	1 (3%)		5 (2%)	..
**Community**								
Intermediate care	..	..	..	2 (13%)	..	..	..	13 (9%)
Other outpatient	..	..	..	1 (6%)	..	..	..	8 (6%)
Own home	..	18 (100%)	..	13 (81%)	..	8 (100%)	..	118 (82%)
Residential home	..	..	..	..	..	..	..	4 (3%)

Supplementary Table S4. Categorical variables (previous exposure) of cases and CD participants groups.

Cases were participants group CCNA positive (presence of *C. difficile* toxin using reference test).
CD term was chosen to describe participants group CTC positive/CCNA negative/SIMOA negative (presence of *C. difficile* without toxin).
Data are number of patients (%) in hospital or community setting with previous exposure prior to the sample being taken, and calculated odds ratio, 95% CI, Chi-squared p values or Fischers exact test p values (when the cell count was less than 5) of cases versus controls and CD versus controls. Antibiotic use and class of antibiotics refers to antibiotics prescribed for treatment or prophylaxis of an infection other than CDI in the preceding 12 weeks. Significant variables are highlighted in bold (p values <0.05 and 95% CI that do not include 1.0).

CCNA: cell-cytotoxin neutralisation assay; CDI: *Clostridioides difficile* infection; CI: confidence intervals; CTC: cell cytotoxigenic culture; n= number of participants exposed to indicated variable. ; N= total number of participants in analysis (with data); na=not able to compute odds ratio due to a value of 0 in one of the groups; p: p values; SIMOA: ultra-sensitive toxin single molecule array.

	Cases	CD	Controls	Cases vs. Controls		CD vs. Controls	
**Variable/Exposure**	**% (n/N)**	**% (n/N)**	**% (n/N)**	**Odds ratio [95% CI]**	**p**	**Odds ratio [95% CI]**	**p**
Contact with healthcare facility (previous 6 months)							
Hospital	**81.0%** (51/63)	62.5% (10/16)	**53.9%** (138/256)	**3.63 [1.80-5.35]**	**<0.0001**	1.43 [0.50-4.04]	0.50
Community	53.0% (9/17)	42.9% (6/14)	38.8% (45/116)	1.78 [0.64-4.94]	0.27	1.18 [0.39-3.64]	0.77
Contact with CDI (previous 3 months)							
Hospital	2.9% (1/35)	0% (0/7)	0.8% (1/118)	3.44 [0.21-56.5]	0.41	na [na]	1.00
Community	0% (0/4)	0% (0/8)	2.0% (1/49)	na [na]	1.00	na [na]	1.00
Contact with infant (previous 3 months)							
Hospital	3.7% (1/27)	0% (0/5)	7.8% (7/90)	0.46 [0.05-3.88]	0.68	na [na]	1.00
Community	33.3% (1/3)	0% (0/4)	10.7% (3/28)	4.17 [0.29-60.9]	0.35	na [na]	1.00
Antibiotic use (previous 12 weeks)							
≥ one antibiotic							
Hospital	**76.1%** (51/67)	75.0% (12/16)	**60.9%** (157/258)	**2.05 [1.11-3.79]**	**0.02**	1.93 [0.61-6.15]	0.26
Community	**46.2%** (6/13)	9.1% (1/11)	**17.7%** (17/96)	**3.98 [1.19-13.4]**	**0.03**	0.47 [0.06-3.88]	0.69
> one antibiotic							
Hospital	43.3% (29/67)	43.8% (7/16)	38.0% (98/258)	1.25 [0.72-2.15]	0.43	1.27 [0.46-3.52]	0.65
Community	**23.1%** (3/13)	0% (0/11)	**2.1%** (2/96)	**14.10 [2.10-94.7]**	**0.01**	na [na]	1.00
> two antibiotics							
Hospital	23.9% (16/67)	18.8% (3/16)	23.6% (61/258)	1.01 [0.54-1.90]	0.97	0.75 [0.21-2.70]	0.77
Community	**23..1%** (3/13)	0% (0/11)	**2.1%** (2/96)	**14.10 [2.10-94.7]**	**0.01**	na [na]	1.00
> three antibiotics							
Hospital	11.9% (8/67)	6.3% (1/16)	15.1% (39/258)	0.76 [0.34-1.72]	0.51	0.37 [0.05-2.92]	0.48
Community	15.4% (2/13)	0% (0/11)	0% (0/96)	na [na]	**0.01**	na [na]	1.00
Class of antibiotic (previous 12 weeks)							
Narrow spectrum penicillins							
Hospital	6.0% (4/67)	6.3% (1/16)	10.9% (28/258)	0.52 [0.18-1.54]	0.23	0.55 [0.07-4.31]	1.00
Community	0% (0/13)	9.1% (1/11)	2.1% (2/96)	na [na]	1.00	4.70 [0.39-56.5]	0.28
Broad spectrum penicillins							
Hospital	20.9% (14/67)	37.5% (6/16)	30.2% (78/258)	0.61 [0.32-1.16]	0.13	1.39 [0.49-3.94]	0.58
Community	**30.8%** (4/13)	0% (0/11)	**5.2%** (5/96)	**8.09 [1.84-35.6]**	**0.01**	na [na]	1.00

Cephalosporins							
Hospital	**31.3%** (21/67)	18.8% (3/16)	**18.6%** (48/258)	**2.00 [1.09-3.65]**	**0.02**	1.01 [0.28-3.68]	1.00
Community	7.7% (1/13)	0% (0/11)	0% (0/96)	na [na]	0.12	na [na]	na
Other betalactams							
Hospital	7.5% (5/67)	0% (0/16)	9.7% (25/258)	0.75 [0.28-2.04]	0.58	na [na]	0.38
Community	7.7% (1/13)	0% (0/11)	1.0% (1/96)	7.92 [0.46-135.0]	0.23	na [na]	1.00
Tetracyclines							
Hospital	4.5% (3/67)	0% (0/16)	1.2% (3/258)	3.98 [0.79-20.2]	0.11	na [na]	1.00
Community	0% (0/13)	0% (0/11)	2.1% (2/96)	na [na]	1.00	na [na]	1.00
Aminoglycosides							
Hospital	6.0% (4/67)	6.3% (1/16)	4.3% (11/258)	1.43 [0.44-4.63]	0.52	1.50 [0.18-12.4]	0.52
Community	7.7% (1/13)	0% (0/11)	1.0% (1/96)	7.92 [0.46-135.0]	0.23	na [na]	1.00
Macrolides							
Hospital	7.5% (5/67)	0% (0/16)	7.8% (20/258)	0.96 [0.35-2.66]	0.94	na [na]	0.62
Community	0% (0/13)	0% (0/11)	1.0% (1/96)	na [na]	1.00	na [na]	1.00
Clindamycin							
Hospital	3.0% (2/67)	0% (0/16)	3.9% (10/258)	0.76 [0.16-3.57]	1.00	na [na]	1.00
Community	0% (0/13)	0% (0/11)	0% (0/96)	na [na]	na	na [na]	na
Sulphonamides							
Hospital	3.0% (2/67)	0% (0/16)	1.9% (5/258)	1.56 [0.30-8.21]	0.64	na [na]	1.00
Community	0% (0/13)	0% (0/11)	0% (0/96)	na [na]	na	na [na]	na
Trimethoprim							
Hospital	3.0% (2/67)	12.5% (2/16)	3.5% (9/258)	0.85 [0.18-4.04]	1.00	3.95 [0.78-20.1]	0.13
Community	0% (0/13)	0% (0/11)	1.0% (1/96)	na [na]	1.00	na [na]	1.00
Antituberculous drugs							
Hospital	1.5% (1/67)	0% (0/16)	0.8% (2/258)	1.94 [0.17-21.7]	0.50	na [na]	1.00
Community	0% (0/13)	0% (0/11)	0% (0/96)	na [na]	na	na [na]	na
Nitroimidazole							
Hospital	6.0% (4/67)	6.3% (1/16)	5.0% (13/258)	1.20 [0.38-3.80]	0.76	1.26 [0.15-10.3]	0.58
Community	7.7% (1/13)	0% (0/11)	1.0% (1/96)	7.92 [0.46-135.0]	0.23	na [na]	1.00
Glycopeptides							
Hospital	6.0% (4/67)	12.5% (2/16)	7.0% (18/258)	0.85 [0.28-2.59]	0.23	1.91 [0.40-9.04]	0.33
Community	7.7% (1/13)	0% (0/11)	0% (0/96)	na [na]	0.12	na [na]	na
Fluoroquinolones, Other quinolones							
Hospital	**25.4%** (17/67)	18.8% (3/16)	**13.2%** (34/258)	**2.24 [1.16-4.33]**	**0.01**	1.52 [0.41-5.61]	0.46
Community	15.4% (2/13)	0% (0/11)	3.1% (3/96)	5.64 [0.85-37.5]	0.11	na [na]	1.00
Other antibiotics							
Hospital	17.9% (12/67)	6.3% (1/16)	13.6% (35/258)	1.39 [0.68-2.85]	0.37	0.43 [0.05-3.32]	0.70
Community	0% (0/13)	0% (0/11)	3.1% (3/96)	na [na]	1.00	na [na]	1.00

Supplementary Table S5. Categorical variables (number and type of co-morbidities) of cases and CD participants groups.

Cases were participants group CCNA positive (presence of *C. difficile* toxin using reference test).
CD term was chosen to describe participants group CTC positive/CCNA negative/SIMOA negative (presence of *C. difficile* without toxin).
Data are number of patients (%) in hospital or community setting with reported co-morbidities, and calculated odds ratio, 95% CI, Chi-squared p values or Fischers exact test p values (when the cell count was less than 5) of cases versus controls and CD versus controls. Significant variables are highlighted in bold (p values <0.05 and 95% CI that do not include 1.0).

CCNA: cell-cytotoxin neutralisation assay; CDI: *Clostridioides difficile* infection; CI: confidence intervals; CTC: cell cytotoxigenic culture; n= number of participants exposed to indicated variable. ; N= total number of participants in analysis (with data); na=not able to compute odds ratio due to a value of 0 in one of the groups; p: p values; SIMOA: ultra-sensitive toxin single molecule array.

	Cases	CD	Controls	Cases vs. Controls		CD vs. Controls	
**Co-morbidities**	**% (n/N)**	**% (n/N)**	**% (n/N)**	**Odds ratio [95% CI]**	**p**	**Odds ratio [95% CI]**	**p**
With ≥ 2 co-morbidities							
Hospital	**59.5%** (44/74)	52.9% (9/17)	**43.0%** (122/284)	**1.95 [1.16-3.28]**	**0.01**	1.49 [0.56-3.98]	0.42
Community	33.3% (4/12)	21.4% (3/14)	15.1% (16/106)	2.81 [0.76-10.5]	0.12	1.53 [0.39-6.12]	0.46
Charlson index score							
0							
Hospital	10.8% (8/74)	11.8% (2/17)	25.4% (72/284)	0.36 [0.16-0.78]	0.01	0.39 [0.09-1.76]	0.26
Community	25.0% (3/12)	64.3% (9/14)	48.1% (51/106)	0.36 [0.09-1.40]	0.13	1.94 [0.61-6.18]	0.26
1							
Hospital	2.7% (2/74)	11.8% (2/17)	4.2% (12/284)	0.63 [0.14-2.88]	0.74	3.02 [0.62-14.7]	0.18
Community	0% (0/12)	7.1% (1/14)	6.6% (7/106)	na [na]	1.00	1.09 [0.12-9.56]	1.00
2							
Hospital	5.4% (4/74)	11.8% (1/17)	8.5% (24/284)	0.62 [0.21-1.84]	0.47	1.44 [0.31-6.69]	0.65
Community	8.3% (1/12)	0% (0/14)	13.2% (14/106)	0.60 [0.07-4.99]	1.00	na [na]	0.37
3							
Hospital	5.4% (4/74)	5.9% (1/17)	9.5% (27/284)	0.54 [0.18-1.61]	0.26	0.60 [0.08-4.66]	1.00
Community	8.3% (1/12)	7.1% (1/14)	6.6% (7/106)	1.29 [0.14-11.4]	0.59	1.09 [0.12-9.56]	1.00
3+							
Hospital	**81.1%** (60/74)	64.7% (11/17)	**62.0%** (176/284)	**2.63 [1.40-4.93]**	**<0.01**	1.13 [0.40-3.13]	1.00
Community	**66.7%** (8/12)	28.6% (4/14)	**32.1%** (34/106)	**4.24 [1.19-15.0]**	**0.03**	0.85 [0.25-2.90]	1.00
4+							
Hospital	**75.7%** (56/74)	58.8% (10/17)	**52.5%** (149/284)	**2.82 [1.58-5.03]**	**<0.0001**	1.29 [0.48-3.50]	0.61
Community	**58.3%** (7/12)	21.4% (3/14)	**25.5%** (27/106)	**4.10 [1.20-14.0]**	**0.04**	0.80 [0.21-3.08]	1.00
Co-morbidities							
Cerebrovascular Disease							
Hospital	**23.0%** (17/74)	5.9% (1/17)	**6.7%** (19/284)	**4.16 [2.04-8.50]**	**<0.0001**	0.87 [0.11-6.93]	1.00
Community	8.3% (1/12)	0% (0/14)	0% (0/106)	na [na]	0.10	na [na]	na
Chronic kidney disease							
Hospital	17.6% (13/74)	23.5% (4/17)	10.6% (30/284)	1.80 [0.89-3.66]	0.10	2.61 [0.80-8.50]	0.11
Community	16.7% (2/12)	0% (0/14)	6.6% (7/106)	2.83 [0.52-15.5]	0.23	na [na]	na
Solid tumour within last 5 years							

Hospital	20.3% (15/74)	5.9% (1/17)	12.7% (36/284)	1.75 [0.90-3.41]	0.10	0.43 [0.06-3.35]	0.71
Community	8.3% (1/12)	7.1% (1/14)	4.7% (5/106)	1.84 [0.20-17.2]	0.48	1.55 [0.17-14.4]	0.53
Pneumonia							
Hospital	**17.6%** (13/74)	0% (0/17)	**5.3%** (15/284)	**3.82 [1.73-8.45]**	**<0.0001**	na [na]	1.00
Community	0% (0/12)	0% (0/14)	0% (0/106)	na [na]	na	na [na]	na
Diabetes without organ damage							
Hospital	14.9% (11/74)	5.9% (1/17)	8.5% (24/284)	1.89 [0.88-4.06]	0.10	0.68 [0.10-5.33]	1.00
Community	16.7% (2/12)	7.1% (1/14)	6.6% (7/106)	2.83 [0.52-15.5]	0.23	1.09 [0.12-9.56]	1.00
UTI							
Hospital	**14.9%** (11/74)	11.8% (2/17)	**6.3%** (18/284)	**2.58 [1.16-5.74]**	**0.02**	1.97 [0.42-9.29]	0.31
Community	8.3% (1/12)	7.1% (1/14)	0% (0/106)	na [na]	0.10	na [na]	0.12
Mental Disorientation							
Hospital	**12.2%** (9/74)	5.9% (1/17)	**4.6%** (13/284)	**2.89 [1.18-7.04]**	**0.03**	1.30 [0.16-10.6]	0.57
Community	0% (0/12)	0% (0/14)	0.9% (1/106)	na [na]	1.00	na [na]	1.00
Peripheral Vascular Disease							
Hospital	13.5% (10/74)	11.8% (2/17)	7.7% (22/284)	1.86 [0.84-4.12]	0.19	1.59 [0.34-7.39]	0.64
Community	8.3% (1/12)	7.1% (1/14)	1.9% (2/106)	4.73 [0.40-56.4]	0.28	4.00 [0.34-47.2]	0.31
Congestive Heart Failure							
Hospital	13.5% (10/74)	11.8% (2/17)	9.5% (27/284)	1.49 [0.69-3.23]	0.29	1.27 [0.28-5.85]	0.67
Community	0% (0/12)	0% (0/14)	2.8% (3/106)	na [na]	1.00	na [na]	1.00
Dementia							
Hospital	8.1% (6/74)	0% (0/17)	5.3% (15/284)	1.58 [0.59-4.23]	0.40	na [na]	1.00
Community	0% (0/12)	7.1% (1/14)	0.9% (1/106)	na [na]	1.00	8.08 [0.48-137.0]	0.22
Myocardial Infarction							
Hospital	10.8% (8/74)	5.9% (1/17)	7.0% (20/284)	1.60 [0.68-3.79]	0.28	0.83 [0.10-6.54]	1.00
Community	0% (0/12)	7.1% (1/14)	1.9% (2/106)	na [na]	1.00	4.00 [0.34-47.2]	0.31
COPD							
Hospital	8.1% (6/74)	5.9% (1/17)	7.7% (22/284)	1.05 [0.41-2.69]	0.92	0.74 [0.09-5.88]	1.00
Community	0% (0/12)	0% (0/14)	0.9% (1/106)	na [na]	1.00	na [na]	1.00
Moderate or severe liver disease							
Hospital	8.1% (6/74)	11.8% (2/17)	6.7% (19/284)	1.23 [0.47-3.20]	0.67	1.86 [0.40-8.74]	0.34
Community	8.3% (1/12)	0% (0/14)	0.9% (1/106)	na [na]	0.10	na [na]	na
Diabetes with organ damage							
Hospital	6.8% (5/74)	17.6% (3/17)	5.6% (16/284)	1.21 [0.43-3.43]	0.78	3.59 [0.94-13.8]	0.08
Community	0% (0/12)	7.1% (1/14)	5.7% (6/106)	na [na]	1.00	1.28 [0.14-11.5]	0.59
Chemotherapy previous 12 weeks							
Hospital	2.7% (2/74)	11.8% (2/17)	7.7% (22/284)	0.33 [0.08-1.44]	0.19	1.59 [0.34-7.39]	0.64
Community	0% (0/12)	7.1% (1/14)	3.8% (4/106)	na [na]	na	1.96 [0.20-18.9]	0.47
Leukaemia within last 5 years							
Hospital	2.7% (2/74)	5.9% (1/17)	2.8% (8/284)	0.96 [0.20-4.61]	1.00	2.16 [0.25-18.3]	0.41
Community	0% (0/12)	0% (0/14)	0% (0/106)	na [na]	na	na [na]	na
Renal RT within 7 days of sample							
Hospital	4.1% (3/74)	0% (0/17)	2.1% (6/284)	1.96 [0.48-8.02]	0.40	na [na]	1.00
Community	0% (0/12)	0% (0/14)	0% (0/106)	na [na]	na	na [na]	na
Transplant							
Hospital	4.1% (3/74)	5.9% (1/17)	4.2% (12/284)	0.96 [0.26-3.49]	1.00	1.42 [0.17-11.6]	0.54

Community	0% (0/12)	0% (0/14)	3.8% (4/106)	na [na]	1.00	na [na]	1.00
Other malignancy within last 5 years							
Hospital	1.4% (1/74)	11.8% (2/17)	3.2% (9/284)	0.42 [0.05-3.36]	0.69	4.07 [0.81-20.5]	0.12
Community	0% (0/12)	0% (0/14)	1.9% (2/106)	na [na]	1.00	na [na]	1.00
Connective Tissue Disease							
Hospital	2.7% (2/74)	5.9% (1/17)	1.1% (3/284)	2.60 [0.43-15.9]	0.28	5.85 [0.58-59.5]	0.21
Community	0% (0/12)	0% (0/14)	0% (0/106)	na [na]	na	na [na]	na
Lymphoma within last 5 years							
Hospital	2.7% (2/74)	0% (0/17)	2.5% (7/284)	1.10 [0.22-5.41]	1.00	na [na]	1.00
Community	0% (0/12)	0% (0/14)	0.9% (1/106)	na [na]	1.00	na [na]	na
Hemiplegia							
Hospital	0% (0/74)	5.9% (1/17)	1.4% (4/284)	na [na]	0.59	4.38 [0.46-41.5]	0.25
Community	8.3% (1/12)	0% (0/14)	0% (0/106)	na [na]	0.10	na [na]	na
Mild liver disease							
Hospital	1.4% (1/74)	5.9% (1/17)	1.4% (4/284)	0.96 [0.11-8.71]	1.00	4.38 [0.46-41.5]	0.25
Community	0% (0/12)	0% (0/14)	1.9% (2/106)	na [na]	1.00	na [na]	1.00
Radiotherapy previous 12 weeks							
Hospital	1.4% (1/74)	0% (0/17)	1.1% (3/284)	1.28 [0.13-12.5]	1.00	na [na]	1.00
Community	0% (0/12)	7.1% (1/14)	3.8% (4/106)	na [na]	na	na [na]	0.12
Cardiac Arrest previous 7 days							
Hospital	1.4% (1/74)	0% (0/17)	0.4% (1/284)	3.88 [0.24-62.7]	0.37	na [na]	1.00
Community	0% (0/12)	0% (0/14)	0% (0/106)	na [na]	na	na [na]	na
AIDS not just HIV positive							
Hospital	0% (0/74)	0% (0/17)	0% (0/106)	na [na]	na	na [na]	na
Community	0% (0/12)	0% (0/14)	0% (0/106)	na [na]	na	na [na]	na
HIV positive							
Hospital	0% (0/74)	0% (0/17)	0% (0/106)	na [na]	na	na [na]	na
Community	0% (0/12)	0% (0/14)	3.8% (4/106)	na [na]	1.00	na [na]	1.00
Peptic Ulcer Disease							
Hospital	0% (0/74)	0% (0/17)	0% (0/106)	na [na]	na	na [na]	na
Community	0% (0/12)	0% (0/14)	0% (0/106)	na [na]	na	na [na]	na
Cystic Fibrosis							
Hospital	0% (0/74)	0% (0/17)	0% (0/106)	na [na]	na	na [na]	na
Community	0% (0/12)	0% (0/14)	0% (0/106)	na [na]	na	na [na]	na
Other comorbidities listed							
Hospital	31.1% (23/74)	23.5% (4/17)	30.6% (87/284)	1.02 [0.59-1.78]	0.94	0.70 [0.22-2.20]	0.54
Community	16.7% (2/12)	14.3% (2/14)	17.9% (19/106)	0.92 [0.19-4.52]	1.00	0.76 [0.16-3.69]	1.00
None							
Hospital	**9.5%** (7/74)	17.6% (3/17)	**29.2%** (83/284)	**0.25 [0.11-0.57]**	**<0.0001**	0.52 [0.15-1.85]	0.41
Community	41.7% (5/12)	71.4% (10/14)	58.5% (62/106)	0.51 [0.15-1.70]	0.27	1.77 [0.52-6.02]	0.35

Supplementary Table S6. Categorical variables (exposure) of SIMOApos participants group.

Categorical variables of patients negative for *C. difficile* infection at the European Coordinating Laboratory but positive for novel SIMOA technology at bioMérieux.
SIMOApos term was chosen to describe participants group SIMOA positive/CCNA negative (presence of *C. difficile* toxin using novel test).
Data are number of patients (%) in hospital or community setting with previous exposure prior to the sample being taken, and calculated odds ratio, 95% CI, Chi-squared p values or Fischers exact test p values (when the cell count was less than 5) of SIMOApos versus controls. Antibiotic use and class of antibiotics refers to antibiotics prescribed for treatment or prophylaxis of an infection other than CDI in the preceding 12 weeks. Significant variables are highlighted in bold (p values <0.05 and 95% CI that do not include 1.0).

CCNA: cell-cytotoxin neutralisation assay; CDI: *Clostridioides difficile* infection; CI: confidence intervals; n= number of participants exposed to indicated variable. ; N= total number of participants in analysis (with data); na=not able to compute odds ratio due to a value of 0 in one of the groups; p: p values; SIMOA: ultra-sensitive toxin single molecule array.

	SIMOApos	Controls	SIMOApos vs. Controls	
**Variable/Exposure**	**% (n/N)**	**% (n/N)**	**Odds ratio [95% CI]**	**p**
Contact with healthcare facility (previous 6 months)				
Hospital	56.7% (17/30)	53.9% (138/256)	1.12 [0.52-2.40]	0.77
Community	83.3% (5/6)	38.8% (45/116)	7.89 [0.89-69.7]	0.04
Contact with CDI (previous 3 months)				
Hospital	8.3% (1/12)	0.8% (1/118)	10.64 [0.62-182.0]	0.18
Community	33.3% (1/3)	2.0% (1/49)	24.0 [1.07-539.1]	0.11
Contact with infant (previous 3 months)				
Hospital	0% (0/10)	7.8% (7/90)	na [na]	1.00
Community	50.0% (1/2)	10.7% (3/28)	8.33 [0.41-170.7]	0.25
Antibiotic use (previous 12 weeks)				
≥ one antibiotic				
Hospital	76.7% (23/30)	60.9% (157/258)	2.11 [0.88-5.11]	0.09
Community	**71.4%** (5/7)	**17.7%** (17/96)	**11.62 [2.08-65.0]**	**0.01**
> one antibiotic				
Hospital	43.3% (13/30)	38.0% (98/258)	1.25 [0.58-2.68]	0.57
Community	**28.6%** (2/7)	**2.1%** (2/96)	**18.80 [2.18-162.5]**	**0.02**
> two antibiotics				
Hospital	33.3% (10/30)	23.6% (61/258)	1.62 [0.72-3.64]	0.24
Community	0% (0/7)	2.1% (2/96)	na [na]	1.00
> three antibiotics				
Hospital	23.3% (7/30)	15.1% (39/258)	1.71 [0.69-4.26]	0.29
Community	0% (0/7)	0% (0/96)	na [na]	na
Class of antibiotic (previous 12 weeks)				
Narrow spectrum penicillins				
Hospital	6.7% (2/30)	10.9% (28/258)	0.59 [0.13-2.60]	0.75
Community	0% (0/7)	2.1% (2/96)	na [na]	1.00
Broad spectrum penicillins				
Hospital	30.0% (9/30)	30.2% (78/258)	0.99 [0.43-2.26]	0.98
Community	28.6% (2/7)	5.2% (5/96)	7.28 [1.12-47.3]	0.07

Cephalosporins				
Hospital	33.3% (10/30)	18.6% (48/258)	2.19 [0.96-4.97]	0.06
Community	14.3% (1/7)	0% (0/96)	na [na]	0.07
Other betalactams				
Hospital	16.7% (5/30)	9.7% (25/258)	1.86 [0.66-5.30]	0.22
Community	14.3% (1/7)	1.0% (1/96)	15.83 [0.88-285.5]	0.13
Tetracyclines				
Hospital	3.3% (1/30)	1.2% (3/258)	2.93 [0.30-29.1]	0.36
Community	0% (0/7)	2.1% (2/96)	na [na]	1.00
Aminoglycosides				
Hospital	6.7% (2/30)	4.3% (11/258)	1.60 [0.34-7.61]	0.63
Community	0% (0/7)	1.0% (1/96)	na [na]	1.00
Macrolides				
Hospital	6.7% (2/30)	7.8% (20/258)	0.85 [0.19-3.83]	1.00
Community	14.3% (1/7)	1.0% (1/96)	15.83 [0.88-285.5]	0.13
Clindamycin				
Hospital	0% (0/30)	3.9% (10/258)	na [na]	0.61
Community	0% (0/7)	0% (0/96)	na [na]	na
Sulphonamides				
Hospital	0% (0/30)	1.9% (5/258)	na [na]	1.00
Community	0% (0/7)	0% (0/96)	na [na]	na
Trimethoprim				
Hospital	3.3% (1/30)	3.5% (9/258)	0.95 [0.12-7.80]	1.00
Community	0% (0/7)	1.0% (1/96)	na [na]	1.00
Antituberculous drugs				
Hospital	0% (0/30)	0.8% (2/258)	na [na]	1.00
Community	0% (0/7)	0% (0/96)	na [na]	na
Nitroimidazole				
Hospital	6.7% (2/30)	5.0% (13/258)	1.35 [0.29-6.27]	0.66
Community	14.3% (1/7)	1.0% (1/96)	15.83 [0.88-285.5]	0.13
Glycopeptides				
Hospital	10.0% (3/30)	7.0% (18/258)	1.48 [0.41-5.36]	0.47
Community	0% (0/7)	0% (0/96)	na [na]	na
Fluoroquinolones, Other quinolones				
Hospital	26.7% (8/30)	13.2% (34/258)	2.40 [0.99-5.81]	0.06
Community	0% (0/7)	3.1% (3/96)	na [na]	1.00
Other antibiotics				
Hospital	20.0% (6/30)	13.6% (35/258)	1.59 [0.61-4.17]	0.41
Community	14.3% (1/7)	3.1% (3/96)	5.17 [0.46-57.5]	0.25

Supplementary Table S7. Categorical variables (number and type of co-morbidities) of SIMOApos participants group.

Categorical variables of patients negative for *C. difficile* infection at the European Coordinating Laboratory but positive for novel SIMOA technology at bioMérieux.
SIMOApos term was chosen to describe participants group SIMOA positive/CCNA negative (presence of *C. difficile* toxin using novel test).
Data are number of patients (%) in hospital or community setting with reported co-morbidities, and calculated odds ratio, 95% CI, Chi-squared p values or Fischers exact test p values (when the cell count was less than 5) of SIMOApos versus controls. Significant variables are highlighted in bold (p values <0.05 and 95% CI that do not include 1.0).

CCNA: cell-cytotoxin neutralisation assay; CDI: *Clostridioides difficile* infection; CI: confidence intervals; n= number of participants exposed to indicated variable. ; N= total number of participants in analysis (with data); na=not able to compute odds ratio due to a value of 0 in one of the groups; p: p values; SIMOA: ultra-sensitive toxin single molecule array.

	SIMOApos	Controls	SIMOApos vs. Controls	
**Co-morbidities**	**% (n/N)**	**% (n/N)**	**OR [95% CI]**	**p**
With ≥ 2 co-morbidities				
Hospital	60.0% (21/35)	43.0% (122/284)	1.99 [0.97-4.08]	0.06
Community	**66.7%** (4/6)	**15.1%** (16/106)	**11.25 [1.90-66.6]**	**0.01**
Charlson index score				
0				
Hospital	11.4% (4/35)	25.4% (72/284)	0.38 [0.13-1.11]	0.07
Community	16.7% (1/6)	48.1% (51/106)	0.22 [0.02-1.91]	0.21
1				
Hospital	8.6% (3/35)	4.2% (12/284)	2.13 [0.57-7.93]	0.22
Community	0% (0/6)	6.6% (7/106)	na [na]	1.00
2				
Hospital	5.7% (2/35)	8.5% (24/284)	0.66 [0.15-2.91]	0.75
Community	16.7% (1/6)	13.2% (14/106)	1.31 [0.14-12.1]	0.58
3				
Hospital	0% (0/35)	9.5% (27/284)	na [na]	0.06
Community	16.7% (1/6)	6.6% (7/106)	2.83 [0.29-27.7.4]	0.37
3+				
Hospital	74.3% (26/35)	62.0% (176/284)	1.77 [0.80-3.93]	0.15
Community	66.7% (4/6)	32.1% (34/106)	4.24 [0.74-24.3]	0.18
4+				
Hospital	**74.3%** (26/35)	**52.5%** (149/284)	**2.62 [1.18-5.78]**	**0.01**
Community	50.0% (3/6)	25.5% (27/106)	2.93 [0.56-15.4]	0.34
Co-morbidities				
Cerebrovascular Disease				
Hospital	14.3% (5/35)	6.7% (19/284)	2.33 [0.81-6.68]	0.16
Community	0% (0/6)	0% (0/106)	na [na]	na
Chronic kidney disease				
Hospital	17.1% (6/35)	10.6% (30/284)	1.75 [0.67-4.56]	0.26
Community	33.3% (2/6)	6.6% (7/106)	7.07 [1.10-45.5]	0.07
Solid tumour within last 5 years				
Hospital	5.7% (2/35)	12.7% (36/284)	0.42 [0.10-1.82]	0.40

Community	16.7% (1/6)	4.7% (5/106)	4.04 [0.39-41.4]	0.29
Pneumonia				
Hospital	11.4% (4/35)	5.3% (15/284)	2.31 [0.72-7.41]	0.14
Community	0% (0/6)	0% (0/106)	na [na]	na
Diabetes without organ damage				
Hospital	14.3% (5/35)	8.5% (24/284)	1.81 [0.64-5.08]	0.34
Community	0% (0/6)	6.6% (7/106)	na [na]	1.00
UTI				
Hospital	11.4% (4/35)	6.3% (18/284)	1.91 [0.61-6.00]	0.28
Community	16.7% (1/6)	0% (0/106)	na [na]	0.06
Mental Disorientation				
Hospital	**17.1%** (6/35)	**4.6%** (13/284)	**4.31 [1.52-12.2]**	**0.01**
Community	16.7% (1/6)	0.9% (1/106)	21.00 [1.14-386.8]	0.11
Peripheral Vascular Disease				
Hospital	11.4% (4/35)	7.7% (22/284)	1.54 [0.50-4.75]	0.51
Community	16.7% (1/6)	1.9% (2/106)	10.40 [0.80-134.9]	0.15
Congestive Heart Failure				
Hospital	8.6% (3/35)	9.5% (27/284)	0.89 [0.26-3.11]	1.00
Community	0% (0/6)	2.8% (3/106)	na [na]	1.00
Dementia				
Hospital	11.4% (4/35)	5.3% (15/284)	2.31 [0.72-7.41]	0.14
Community	16.7% (1/6)	0.9% (1/106)	21.00 [1.14-386.8]	0.11
Myocardial Infarction				
Hospital	2.9% (1/35)	7.0% (20/284)	0.39 [0.05-2.99]	0.49
Community	0% (0/6)	1.9% (2/106)	na [na]	1.00
COPD				
Hospital	8.6% (3/35)	7.7% (22/284)	1.12 [0.32-3.94]	0.75
Community	0% (0/6)	0.9% (1/106)	na [na]	1.00
Moderate or severe liver disease				
Hospital	5.7% (2/35)	6.7% (19/284)	0.85 [0.19-3.79]	1.00
Community	0% (0/6)	0.9% (1/106)	na [na]	na
Diabetes with organ damage				
Hospital	2.9% (1/35)	5.6% (16/284)	0.49 [0.06-3.83]	0.71
Community	0% (0/6)	5.7% (6/106)	na [na]	1.00
Chemotherapy previous 12 weeks				
Hospital	8.6% (3/35)	7.7% (22/284)	1.12 [0.32-3.94]	0.75
Community	16.7% (1/6)	3.8% (4/106)	5.10 [0.48-54.5]	0.25
Leukaemia within last 5 years				
Hospital	8.6% (3/35)	2.8% (8/284)	3.23 [0.82-12.8]	0.11
Community	0% (0/6)	0% (0/106)	na [na]	na
Renal RT within 7 days of sample				
Hospital	2.9% (1/35)	2.1% (6/284)	1.36 [0.16-11.70]	0.56
Community	0% (0/6)	0% (0/106)	na [na]	na
Transplant				
Hospital	2.9% (1/35)	4.2% (12/284)	0.67 [0.08-5.29]	1.00
Community	0% (0/6)	3.8% (4/106)	na [na]	1.00

Other malignancy within last 5 years				
Hospital	5.7% (2/35)	3.2% (9/284)	1.85 [0.38-8.94]	0.35
Community	0% (0/6)	1.9% (2/106)	na [na]	1.00
Connective Tissue Disease				
Hospital	2.9% (1/35)	1.1% (3/284)	2.76 [0.28-27.2]	0.37
Community	0% (0/6)	0% (0/106)	na [na]	na
Lymphoma within last 5 years				
Hospital	0% (0/35)	2.5% (7/284)	na [na]	1.00
Community	0% (0/6)	0.9% (1/106)	na [na]	1.00
Hemiplegia				
Hospital	2.9% (1/35)	1.4% (4/284)	2.06 [0.22-19.0]	0.44
Community	0% (0/6)	0% (0/106)	na [na]	na
Mild liver disease				
Hospital	0% (0/35)	1.4% (4/284)	na [na]	1.00
Community	0% (0/6)	1.9% (2/106)	na [na]	1.00
Radiotherapy previous 12 weeks				
Hospital	0% (0/35)	1.1% (3/284)	na [na]	1.00
Community	0% (0/6)	3.8% (4/106)	na [na]	na
Cardiac Arrest previous 7 days				
Hospital	0% (0/35)	0.4% (1/284)	na [na]	1.00
Community	0% (0/6)	0% (0/106)	na [na]	na
AIDS not just HIV positive				
Hospital	2.9% (1/35)	0% (0/106)	na [na]	0.11
Community	0% (0/6)	0% (0/106)	na [na]	na
HIV positive				
Hospital	0% (0/35)	0% (0/106)	na [na]	na
Community	0% (0/6)	3.8% (4/106)	na [na]	1.00
Peptic Ulcer Disease				
Hospital	0% (0/35)	0% (0/106)	na [na]	na
Community	0% (0/6)	0% (0/106)	na [na]	na
Cystic Fibrosis				
Hospital	0% (0/35)	0% (0/106)	na [na]	na
Community	0% (0/6)	0% (0/106)	na [na]	na
Other comorbidities listed				
Hospital	**48.6%** (17/35)	**30.6%** (87/284)	**2.14 [1.05-4.35]**	**0.03**
Community	16.7% (1/6)	17.9% (19/106)	0.92 [0.10-8.30]	1.00
None				
Hospital	20.0% (7/35)	29.2% (83/284)	0.61 [0.25-1.44]	0.25
Community	33.3% (2/6)	58.5% (62/106)	0.36 [0.06-2.02]	0.40
